# Causal effect of central obesity on left ventricular structure and function in preserved EF population: A Mendelian randomization study

**DOI:** 10.3389/fcvm.2022.1103011

**Published:** 2023-01-09

**Authors:** Yue Gao, Jiaxin Zeng, Fengwei Zou, Xinwei Zhang, Zhiyong Qian, Yao Wang, Xiaofeng Hou, Jiangang Zou

**Affiliations:** ^1^Department of Cardiology, The First Affiliated Hospital of Nanjing Medical University, Nanjing, China; ^2^Montefiore Medical Center, New York, NY, United States; ^3^Key Laboratory of Targeted Intervention of Cardiovascular Disease, Collaborative Innovation Center for Cardiovascular Disease Translational Medicine, Nanjing Medical University, Nanjing, China

**Keywords:** central obesity, left ventricular structure and function, causal association, Mendelian randomization, waist-to-hip ratio

## Abstract

**Background:**

Observational studies have shown that central obesity is associated with adverse cardiac structure and function. However, causal association between central obesity and left ventricular (LV) structure and function in preserved ejection fraction (EF) population is still uncertain.

**Methods:**

Genome-wide association studies summary data of waist circumference adjusted for body mass index (WCadjBMI) and waist-to-hip ratio adjusted for body mass index (WHRadjBMI) were selected as instrumental variables from the Genetic Investigation of Anthropometric Traits (GIANT) Consortium (*n* = 224,459). Outcome datasets for LV parameters including LV end-diastolic volume (LVEDV), LV end-systolic volume (LVESV), LV ejection fraction (LVEF), LV mass (LVM), and LV mass-to-end-diastolic volume ratio (LVMVR) were obtained from the participants without prevalent myocardial infarction or heart failure (LVEF ≥ 50%) in UK Biobank Cardiovascular Magnetic Resonance sub-study (*n* = 16,923). Two-sample Mendelian randomization (MR) was performed with the inverse-variance weighted (IVW) method as the primary estimate and with the weighted median and MR-Egger as the supplemental estimates. Sensitivity analysis was used to assess the heterogeneity and pleiotropic bias in the MR results.

**Results:**

In the IVW analysis, every 1-standard deviation (SD) higher WHRadjBMI was significantly associated with higher LVMVR (β = 0.4583; 95% confidence interval [CI]: 0.2921 to 0.6244; *P* = 6.418 × 10^–8^) and lower LVEDV (β = –0.2395; 95% CI: –0.3984 to –0.0807; *P* = 0.0031) after Bonferroni adjustment. No heterogeneity and horizontal pleiotropy were detected in the analysis. No association of WCadjBMI was found with LVEF, LVEDV, LVESV, LVM, or LVMVR.

**Conclusion:**

Our findings provide evidence of significant causal association between WHRadjBMI and adverse changes in LV structure and function in preserved EF population.

## Introduction

Heart failure (HF) is a global health epidemic and burden, leading to increased morbidity and mortality (1). The total number of HF patients is still rising, especially with an alarming trend in young population, possibly related to the prevalence of obesity (2). The link between obesity and the risk of HF was first confirmed in Framingham Heart Study and is stronger than those for other types of cardiovascular disease (3, 4). Obesity predicts HF with preserved ejection fraction (HFpEF) but not HF with reduced ejection fraction (HFrEF) among those who develop HF (5, 6), and more than 80% of HFpEF patients in the US are overweight or obese (7).

Obesity is commonly defined by body mass index (BMI) to describe the total adipose accumulation. However, regional fat distribution may play a pivotal role in the development of HF (6). Central obesity, usually measured by waist circumference (WC) or waist-to-hip ratio (WHR), has a more prevalence in patients with HFpEF and a more association with increased risk of HF hospitalization or death than general obesity (8–10). Adverse cardiac remodeling, in the form of structural and functional abnormalities of the heart (mainly left ventricular, LV) in response to various stimuli, is associated with the development of HF (11). Observational studies have shown that central obesity is associated with cardiac remodeling independent of BMI (8, 12). Eschalier et al. have observed that cardiac concentric remodeling was associated with central obesity in asymptomatic and normotensive healthy subjects with central obesity (13). However, due to potential residual confounding and reverse causality in observational studies, whether a causal relationship exists between central obesity and cardiac remodeling and dysfunction in preserved ejection fraction (EF) population remains unclear.

Mendelian randomization (MR) is an epidemiological technique capable of elucidating causal estimate of exposures to outcomes, using genetic variants as instrumental variables (IVs) (14). As genetic variants are randomly allocated at conception, genetically predicted exposure in MR is minimally affected by confounders or reverse causation. We conducted a MR study to investigate the potential effects of genetic liability to central obesity measured by WC and WHR on LV structure and function in preserved EF population.

## Materials and methods

### Study design

**FIGURE 1:**
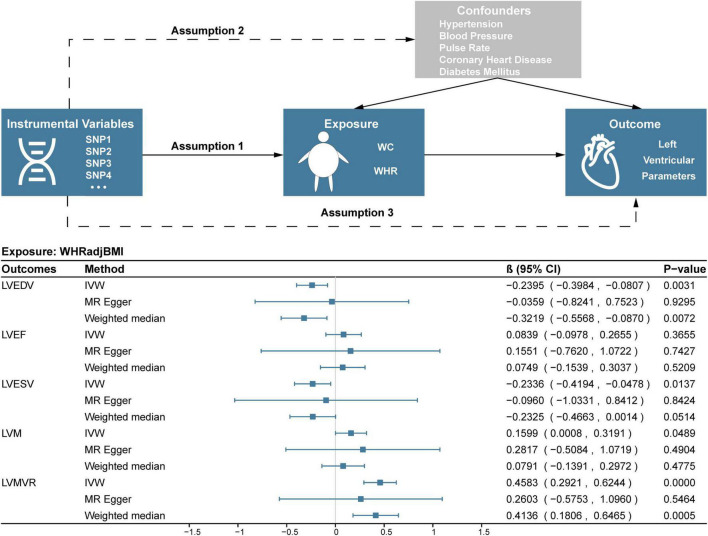
Study design and results of MR analysis of the association between WHRadjBMI and left ventricular parameters. SNP, single nucleotide polymorphism; WC, waist circumference; WHR, waist-to-hip ratio; WHRadjBMI, waist-to-hip ratio adjusted for body mass index; LVEF, left ventricular ejection fraction; LVEDV, left ventricular end-diastolic volume; LVESV, left ventricular end-systolic volume; LVM, left ventricular mass; LVMVR, left ventricular mass-to-end-diastolic volume ratio.

As shown in Figure 1, a two-sample MR model was used to evaluate the causal effect of central obesity on left ventricular structure and function. The study was based on summary-level data on WC adjusted for BMI (WCadjBMI), WHR adjusted for BMI (WHRadjBMI), and parameters of left ventricular structure and function from the published genome-wide association studies (GWASs). The MR design fulfilled three assumptions: (1) genetic instruments are closely related to exposures; (2) genetic instruments are independent of confounders; (3) genetic instruments only affect outcomes *via* the exposures of interest (15).

### Genetic instrument selection

Single nucleotide polymorphisms (SNPs) as instrumental variables associated with WCadjBMI and WHRadjBMI at the genome-wide significance level (*P* < 5 × 10^–8^) were obtained in 224,459 European individuals from the Genetic Investigation of Anthropometric Traits (GIANT) Consortium (Table 1) (16). After estimating linkage disequilibrium (LD *r*^2^ < 0.001, LD distance > 10,000 kb) among the SNPs based on the 1000 Genomes European reference panel (17), we extracted 65 SNPs and 38 SNPs that genetically predicted WCadjBMI and WHRadjBMI, respectively. All SNPs were not associated with the outcome. In order to avoid specific confounders (e.g., hypertension, coronary heart disease, blood pressure, pulse rate, and diabetes mellitus), we excluded 10 of 65 SNPs (rs1344674, rs7684221, rs2071449, rs12656497, rs806794, rs6905288, rs606452, rs3786897, rs459193, rs849140) and 7 of 38 SNPs (rs2071449, rs7705502, rs7759742, rs998584, rs2820443, rs1128249, rs459193) with a threshold of *P* < 5 × 10^––8^ based on the Phenoscanner database^1^ (18). Additionally, one SNP (rs16957304) associated with WCadjBMI missing in the outcome datasets was excluded for its limited influence on the results with a small proportion. In the end, fifty-two WCadjBMI and thirty WHRadjBMI related independent SNPs (Supplementary Tables 1, 2) were considered as instruments for main MR analyses after removing palindromic SNPs (WCadjBMI: rs7162542, rs984222; WHRadjBMI: rs2276824) (19). *F* statistics for the SNPs were calculated to evaluate the strength of the instrument variables (20).

**TABLE 1:** Characteristics of the GWASs used in the present study.

Phenotype	Sample size	PMID	Consortium	Ancestry	Adjusted covariates
**Exposure**					
WC	231,353	25673412	GIANT	European	Age, age-squared, study-specific covariates and BMI
WHR	210,082				
**Outcome**					
LVEF	16,923	31554410	UK Biobank	European	Age, sex, height, weight, systolic blood pressure, phenotype-derivation method, array type, and imaging center
LVEDV	16,920				
LVESV	16,920				
LVM	16,920				
LVMVR	16,884				

GWAS, genome-wide association studies; WC, waist circumference; WHR, waist-to-hip ratio; LVEF, left ventricular ejection fraction; LVEDV, left ventricular end-diastolic volume; LVESV, left ventricular end-systolic volume; LVM, left ventricular mass; LVMVR, left ventricular mass-to-end-diastolic volume ratio; GIANT, Genetic Investigation of Anthropometric Traits Consortium.

### Data source for outcomes

The outcomes of the study were selected based on the GWAS conducted by Aung et al. comprising 16,923 European UK Biobank participants without prevalent myocardial infarction or heart failure (LVEF in every participant ≥ 50%) to identify the genetic loci for 6 relevant cardiac magnetic resonance (CMR)-derived LV imaging phenotypes, including LV end-diastolic volume (LVEDV), LV end-systolic volume (LVESV), LV stroke volume (LVSV), LV ejection fraction (LVEF), LV mass (LVM), and LV mass-to-end-diastolic volume ratio (LVMVR) (21). The GWAS analysis was adjusted for age, sex, height, weight, systolic blood pressure, phenotype-derivation method, array type, and imaging center. We used the summary-level data of 5 parameters (except LVSV) as the outcomes in our study.

### Mendelian randomization analyses

The random effects inverse-variance weighed (IVW) was used as the main MR method in our study while MR-Egger, weighted median and MR-PRESSO were also performed for more robust estimates. IVW analysis estimates the effect of each SNP on the outcome by calculating the Wald ratio and performs a meta-analysis for the combined causal effect with the inverse variance of SNPs as weights (22). MR-Egger provides the estimate with adjustment for horizontal pleiotropy based on the assumption that the effect of the genetic instruments is uncorrected with any pleiotropic effect (23). Weighted median provides consistent estimates based on the assumption but requires more than 50% of weight from valid genetic instruments (24). MR-PRESSO method can detect and correct outlier SNPs and provide estimates after removing outliers (25). For further sensitivity analyses, we conduct the Cochran’s Q test to assess the heterogeneity, the MR-Egger intercept test to analyze the horizontal pleiotropy and leave-one-out analysis to detect high influence points (23, 26, 27). We calculated MR power using a wed-based tool^2^ according to Burgess’s method (28).

### Statistical analysis

All analyses were performed in R software (version 4.2.1) using the packages Two SampleMR (version 0.5.6) and MR-PRESSO (version 1.0). The association with *P*-value < 0.005 (0.05/10) was considered a significant association, and a *P*-value < 0.05 and ≥ 0.005 was regarded as nominally significant after Bonferroni adjustment.

## Results

A total of 52 and 30 SNPs genetically associated with WCadjBMI and WHRadjBMI were enrolled to analyze before removing outliers, respectively (Supplementary Tables 1, 2). All the *F* statistics (Supplementary Tables 1, 2) for instruments were over 10, indicating a good strength of each instrument.

### Association of WCadjBMI with LV parameters

As shown in Table 2, no causal associations were found between WCadjBMI and LV parameters. Results of heterogeneity and pleiotropy tests were shown in Table 3, and there was no pleiotropy in the analysis. Scatter, leave-one-out and funnel plots were reported in the Supplementary Figures 1–6. One outlier (rs7970350) was identified with MR-PRESSO when exploring the association between WCadjBMI and LVEDV. After removing the outlier, every 1-SD increase in genetic liability to WCadjBMI was nominally significantly associated with lower LVEDV in the IVW analysis (β = –0.1718, 95% confidence interval [CI] –0.3311 to –0.0125; *P* = 0.0345) without horizontal pleiotropy (Supplementary Tables 3, 4).

**TABLE 2:** MR analysis of the association between WCadjBMI and left ventricular parameters.

			IVW		MR Egger		Weighted median	
Exposure	No. of SNPs	Outcome	β ± SE	*P*-value	β ± SE	*P*-value	β ± SE	*P*-value
WCadjBMI	52	LVEF	0.0490 ± 0.0725	0.4997	0.1052 ± 0.3092	0.7350	0.0038 ± 0.0993	0.9695
		LVEDV	−0.1427 ± 0.0875	0.1029	0.2687 ± 0.3684	0.4691	−0.1758 ± 0.1005	0.0803
		LVESV	−0.1206 ± 0.0800	0.1317	0.1513 ± 0.3388	0.6572	−0.1347 ± 0.0959	0.1600
		LVM	−0.0700 ± 0.0688	0.3090	0.3566 ± 0.2868	0.2195	−0.0442 ± 0.0921	0.6314
		LVMVR	0.0634 ± 0.0769	0.4102	−0.0831 ± 0.3275	0.8008	0.0041 ± 0.1024	0.9678

WCadjBMI, waist circumference adjusted for body mass index; IVW, inverse-variance weighted; LVEF, left ventricular ejection fraction; LVEDV, left ventricular end-diastolic volume; LVESV, left ventricular end-systolic volume; LVM, left ventricular mass; LVMVR, left ventricular mass-to-end-diastolic volume ratio.

**TABLE 3:** Heterogeneity and horizontal pleiotropy test of the associations between WCadjBMI and WHRadjBMI and left ventricular parameters.

		IVW		MR-egger				
Exposure	Outcome	Cochran’s Q	*P*-value	Cochran’s Q	*P*-value	Intercept	SE	*P*-value for intercept
WCadjBMI	LVEF	63.31	0.1155	63.27	0.0985	–0.0015	0.0078	0.8521
	LVEDV	93.29	0.0003	90.89	0.0004	–0.0107	0.0093	0.2558
	LVESV	77.88	0.0090	76.83	0.0087	–0.0071	0.0086	0.4129
	LVM	57.40	0.2501	54.83	0.2966	–0.0111	0.0073	0.1321
	LVMVR	71.65	0.0298	71.34	0.0254	0.0038	0.0083	0.6473
WHRadjBMI	LVEF	37.47	0.1346	37.44	0.1095	–0.0020	0.0129	0.8776
	LVEDV	27.63	0.5380	27.36	0.4988	–0.0057	0.0111	0.6092
	LVESV	39.66	0.0896	39.54	0.0726	–0.0039	0.0132	0.7710
	LVM	20.48	0.8774	20.38	0.8501	–0.0034	0.0111	0.7600
	LVMVR	31.53	0.3410	31.28	0.3049	0.0056	0.0117	0.6392

WCadjBMI, waist circumference adjusted for body mass index; WHRadjBMI, waist-to-hip ratio adjusted for body mass index; IVW, inverse-variance weighted; LVEF, left ventricular ejection fraction; LVEDV, left ventricular end-diastolic volume; LVESV, left ventricular end-systolic volume; LVM, left ventricular mass; LVMVR, left ventricular mass-to-end-diastolic volume ratio.

### Association of WHRadjBMI with LV parameters

In the IVW analysis, one-SD genetically determined increase in WHRadjBMI was significantly associated with lower LVEDV (β = –0.2395, 95% CI –0.3984 to –0.0807; *P* = 0.0031) and higher LVMVR (β = 0.4583, 95% CI 0.2921 to 0.6244; *P* < 0.0001). Additionally, every 1-SD increase in genetic liability to WHRadjBMI was nominally significantly associated with lower LVESV (β = –0.2336, 95% CI –0.4194 to –0.0478; *P* = 0.0137) and higher LVM (β = 0.1599, 95% CI 0.0008 to 0.3191; *P* = 0.0489). Weighted median and MR-Egger analyses also showed similar associations (Figure 1). No evidence supported that genetic liability to WHRadjBMI was associated with LVEF (Figure 1). Several sensitivity analyses showed no heterogeneity or pleiotropy in Table 3 and no outlier was found using MR-PRESSSO. Scatter, leave-one-out and funnel plots were reported in the Supplementary Figures 1–6.

## Discussion

This two-sample MR study described the major finding that every 1-SD genetically determined increased WHR is causally associated with lower LVEDV and higher LVMVR in preserved EF population. The findings were robust based on different kinds of MR methods and sensitivity analyses.

Multiple population-based studies have observed that both general obesity and central obesity are major risk factors for the development of HF (3, 29–32). Several studies demonstrated general obesity is causally associated with HF (33–35). Our findings are consistent with the prior studies, while we focused on the causal effect of central obesity measured by anthropometrics on LV morphology and function in preserved EF population. With the increasing evidence in HFpEF pathogenesis, obesity has been considered as a primary and direct cause of HFpEF, instead of comorbid bystander, mediated *via* other metabolic syndromes (36). A recent study describing LV structural characteristics of HFpEF across different LVEF reported that individuals with higher LVEF (> 60%) presented more concentric remodeling and diastolic/systolic stiffness, while those with lower LVEF (50–60%) were more characterized by eccentric myocardial remodeling and a higher amount of myocardial fibrosis similar to HFrEF (37). This suggested that there is a dynamic variation of phenotypes with adverse changes in cardiac structure and function. Although we did not investigate the causal association of WC and WHR with the risk of both HFpEF and HFrEF due to a lack of certain GWAS summaries, LV remodeling with preserved LVEF is a critical preclinical characteristic.

Obesity cardiomyopathy increasingly attracts more attention as epidemiological, clinical, and experimental evidence support the existence of this unique disease entity, which develops independent of coronary heart disease, hypertension, and other cardiovascular diseases (38). Alterations of LV structure and function have been noted in obesity with the use of echocardiography and magnetic resonance imaging during observational studies (39–41). The Dallas Heart Study observed the impact of longitudinal changes in adiposity on concentric left ventricular remodeling (42). We further confirmed the causal association of WHR with lower LVEDV and higher LVMVR in this two-sample MR study. This also shed light on the causal relationship of the distribution of adipose tissue on LV remodeling.

Diastolic dysfunction has been reported in obese individuals without meeting diagnosis of HF (38). Yagmur et al. found transmitral deceleration time, isovolumetric relaxation time, and peak late diastolic tissue doppler velocity values, which reflect LV diastolic function, were significantly higher in obese individuals compared with normal weight subjects without significant difference in LVEF between groups (43). Similarly in children, a cross-sectional study found higher ratio of transmitral early diastolic filling velocity to septal peak early diastolic myocardial velocity (E/e’) without left ventricular hypertrophy in obese patients (44). LVMVR is a good parameter to assess the diastolic performance (45) and we further confirmed the causal effect of central obesity on diastolic dysfunction reflected by higher LVMVR with increasing WHR in our study. However, we did not find any difference in LVEF as WHR and WC increased. On one hand, the outcome data were all from those with preserved LVEF, which means the systolic function might not be impaired. On the other hand, this might suggest that central obesity mainly affects cardiac diastolic function in the early disease process. The CARDIA study, a multi-center prospective study that enrolled 5,115 white and black men and women aged between 18 and 30, found longstanding obesity for more than 20 years is associated with overtly impaired LV systolic function as well (46).

Animal studies also observed improvement in cardiac function after weight and fat mass reduction, although Partington et al. emphasized an improvement in left ventricular hypertrophy rather than diastolic dysfunction possibly due to a short follow-up duration (47, 48). Benefits of general obesity control on left ventricular diastolic function was reported in humans (49, 50). Sundström et al. demonstrated that bariatric surgery leads to a lower incidence of HF with a hazard ratio of 0.54 compared with intensive lifestyle treatment (51). Nevertheless, no further subgroup analyses were performed based HF phenotype, such as HFpEF and HFrEF. For central obesity, the Look AHEAD study found that decline in WC was significantly associated with lower risk of HFpEF in adults with type 2 diabetes (52). The Utah obesity study also suggested reverse cardiac remodeling and improved cardiac function along with significant reductions in WC after gastric bypass surgery (53). Recently, the role of WHR, another easily available measurement of central obesity, in cardiovascular diseases draws increasing attention (54). Our data suggested WHR was causally associated with more LV parameters than WC. WHR, affected by both gluteofemoral subcutaneous adipose tissue and abdominal visceral adipose tissue, is considered more accurate to evaluate central obesity than WC for those with large body size. Individuals with large body size without central obesity might be misdiagnosed due to the high WC (55). Yet, considering that both of WHR and WC are easily available, it is better to assess central obesity comprehensively *via* measuring both WHR and WC.

## Limitations

Our two-sample MR analysis had several strengths: (1) The MR method could minimize confounding and reverse causality compared with conventional observational studies; (2) all summary data were based on the population from European descent, which effectively mitigated the bias of population stratification; (3) to reduce pleiotropic effects, we selected the IVs through a rigorous procedure and no significant pleiotropy was observed *via* MR-Egger intercept test and MR-PRESSO analysis. Our study had certain limitations: (1) we could not further explore the causal association upon gender, age, etc. *via* subgroup analyses because our study used the summary-level data rather than individual-level data; (2) our study was confined to individuals of European descent, which limits the generalizability of the findings to other populations; (3) central obesity in our study did not include measurement of visceral fat, which is closely associated with cardiac structure and function as well; (4) all exposure data from the individuals with preserved LVEF in UK Biobank led to healthy worker effect.

## Conclusion

In summary, this MR study supports the genetic causality between WHRadjBMI and adverse changes in LV structure and function in preserved EF population. Our findings may strengthen our understanding of the critical role of central obesity in cardiac remodeling.

## Data availability statement

Data on WCadjBMI and WHRadjBMI have been contributed by the GIANT investigators and have been downloaded from the IEU OpenGWAS Project (GWAS ID: WCadjBMI, ieu-a-67 and WHRadjBMI, ieu-a-79) at: https://gwas.mrcieu.ac.uk/. Data on LV parameters were contributed by the UK Biobank investigators and have been downloaded from https://www.ebi.ac.uk/gwas/home.

## Ethics statement

Ethical review and approval was not required for the study on human participants in accordance with the local legislation and institutional requirements. Written informed consent for participation was not required for this study in accordance with the national legislation and the institutional requirements.

## Author contributions

JZo and YG designed the study. YG and JZe performed the data analysis and drafted the manuscript. FZ, XZ, ZQ, YW, and XH revised the manuscript. JZo supervised the study and acquired funding for the work. All authors have read and agreed to the published version of the manuscript.

## Footnotes

1
http://www.phenoscanner.medschl.cam.ac.uk/

2
https://sb452.shinyapps.io/power/
