# Supplementary material for: Causal effect of central obesity on left ventricular structure and function in preserved EF population: A Mendelian randomization study
Source: Front Cardiovasc Med. 2023 Jan 9;9:1103011. doi: 10.3389/fcvm.2022.1103011 (PMC9869108; doi:10.3389/fcvm.2022.1103011)
Supplement: Supplementary file 1 [file Data_Sheet_1.docx]

**Supplementary Materials**

Table S1. 52 SNPs represented genetically predicted WCadjBMI.

| **SNP** | **Postion** | **Effect allele** | **Other allele** | **Effect allele frequency** | **β** | ***P*-value** | **SE** | **Samplesize** | **R2** | **F** |
| --- | --- | --- | --- | --- | --- | --- | --- | --- | --- | --- |
| rs10041657 | chr 5:108152428 | A | G | 0.2167 | 0.025 | 2.9×10^-10^ | 0.004 | 230824 | 0.00021218 | 39.0625 |
| rs10748826 | chr 10:104354804 | C | T | 0.4224 | -0.023 | 3.4×10^-10^ | 0.0037 | 195019 | 0.00025813 | 38.641344 |
| rs11144688 | chr 9:78542286 | A | G | 0.0917 | -0.034 | 1.9×10^-08^ | 0.006 | 214513 | 0.00019257 | 32.1111111 |
| rs11205277 | chr 1:149892872 | G | A | 0.3898 | 0.027 | 1.3×10^-13^ | 0.0036 | 215898 | 0.00034679 | 56.25 |
| rs12207675 | chr 6:76237741 | C | T | 0.1333 | 0.031 | 3.1×10^-09^ | 0.0052 | 231322 | 0.00022205 | 35.5399408 |
| rs12317176 | chr 12:124404718 | C | T | 0.3833 | -0.02 | 5.9×10^-09^ | 0.0035 | 230924 | 0.0001891 | 32.6530612 |
| rs12330322 | chr 3:72455355 | T | C | 0.225 | -0.022 | 3.2×10^-08^ | 0.004 | 229015 | 0.0001688 | 30.25 |
| rs12493901 | chr 3:171922055 | A | G | 0.4583 | -0.021 | 8.3×10^-10^ | 0.0034 | 230668 | 0.00021897 | 38.1487889 |
| rs12608504 | chr 19:18389135 | G | A | 0.6583 | -0.02 | 1.5×10^-08^ | 0.0036 | 228998 | 0.00017995 | 30.8641975 |
| rs12679556 | chr 8:72514228 | G | T | 0.2083 | 0.026 | 1.3×10^-11^ | 0.0039 | 225056 | 0.00022296 | 44.4444444 |
| rs12700664 | chr 7:25875151 | T | C | 0.4833 | -0.019 | 2.4×10^-08^ | 0.0034 | 227892 | 0.0001803 | 31.2283737 |
| rs12991495 | chr 2:25486770 | C | T | 0.325 | -0.028 | 6.2×10^-14^ | 0.0037 | 229964 | 0.00034398 | 57.2680789 |
| rs13083798 | chr 3:52649748 | G | A | 0.4583 | -0.02 | 3.4×10^-09^ | 0.0034 | 230391 | 0.00019861 | 34.6020761 |
| rs13210323 | chr 6:35005084 | C | A | 0.275 | -0.022 | 1.4×10^-08^ | 0.0038 | 231199 | 0.000193 | 33.5180055 |
| rs17451107 | chr 3:156797609 | C | T | 0.375 | -0.026 | 1.3×10^-13^ | 0.0036 | 227636 | 0.00031688 | 52.1604938 |
| rs1776897 | chr 6:34195011 | T | G | 0.925 | -0.061 | 5.6×10^-20^ | 0.0067 | 197374 | 0.00051629 | 82.8915126 |
| rs1812175 | chr 4:145574844 | G | A | 0.8083 | 0.033 | 4.0×10^-13^ | 0.0045 | 230433 | 0.00033748 | 53.7777778 |
| rs2047937 | chr 16:49864791 | T | C | 0.5333 | -0.019 | 4.7×10^-08^ | 0.0034 | 231009 | 0.0001797 | 31.2283737 |
| rs2052670 | chr 2:66218481 | G | A | 0.4083 | 0.02 | 1.5×10^-08^ | 0.0035 | 231210 | 0.00019327 | 32.6530612 |
| rs2124969 | chr 2:160989486 | C | T | 0.4083 | 0.02 | 7.1×10^-09^ | 0.0034 | 231284 | 0.00019327 | 34.6020761 |
| rs2160077 | chr 14:92428410 | A | G | 0.3917 | -0.018 | 4.5×10^-08^ | 0.0033 | 231255 | 0.0001544 | 29.7520661 |
| rs2179129 | chr 22:29450923 | G | A | 0.45 | -0.019 | 2.6×10^-08^ | 0.0034 | 228844 | 0.0001787 | 31.2283737 |
| rs2214442 | chr 7:20392787 | G | A | 0.4417 | 0.026 | 3.9×10^-09^ | 0.0045 | 152053 | 0.0003334 | 33.382716 |
| rs2274432 | chr 1:184020945 | A | G | 0.3729 | 0.025 | 1.7×10^-12^ | 0.0036 | 227843 | 0.00029231 | 48.2253086 |
| rs2638953 | chr 12:28534415 | C | G | 0.6333 | 0.024 | 6.5×10^-11^ | 0.0036 | 228074 | 0.00026753 | 44.4444444 |
| rs272869 | chr 5:131677997 | G | A | 0.6583 | 0.021 | 6.7×10^-10^ | 0.0034 | 229935 | 0.0001984 | 38.1487889 |
| rs2745353 | chr 6:127452935 | T | C | 0.55 | 0.029 | 7.9×10^-19^ | 0.0033 | 231143 | 0.0004163 | 77.2268136 |
| rs3760318 | chr 17:29247715 | A | G | 0.3583 | -0.021 | 9.0×10^-10^ | 0.0035 | 228998 | 0.00020279 | 36 |
| rs3791679 | chr 2:56096892 | G | A | 0.275 | -0.035 | 2.1×10^-19^ | 0.0039 | 228968 | 0.00048847 | 80.539119 |
| rs395962 | chr 6:105397418 | G | T | 0.6333 | -0.029 | 1.3×10^-15^ | 0.0036 | 231306 | 0.00039061 | 64.8919753 |
| rs4239436 | chr 18:20731930 | G | A | 0.7417 | 0.04 | 1.0×10^-22^ | 0.0041 | 229607 | 0.00061306 | 95.1814396 |
| rs4246302 | chr 15:100687967 | G | A | 0.3333 | 0.022 | 5.7×10^-09^ | 0.0037 | 227205 | 0.0002151 | 35.3542732 |
| rs4542783 | chr 19:8642160 | C | T | 0.3879 | -0.023 | 1.7×10^-08^ | 0.004 | 170860 | 0.0002512 | 33.0625 |
| rs4567683 | chr 15:100778837 | G | A | 0.7167 | -0.022 | 7.7×10^-09^ | 0.0038 | 228589 | 0.00019654 | 33.5180055 |
| rs473902 | chr 9:98256235 | G | T | 0.0583 | -0.049 | 4.3×10^-12^ | 0.0071 | 204544 | 0.00026364 | 47.6294386 |
| rs4886782 | chr 15:74228810 | A | G | 0.2667 | -0.024 | 6.0×10^-12^ | 0.0036 | 228446 | 0.0002253 | 44.4444444 |
| rs6556301 | chr 5:176527577 | T | G | 0.375 | 0.028 | 1.8×10^-12^ | 0.0039 | 191245 | 0.0003675 | 51.5450362 |
| rs6751657 | chr 2:33405151 | C | T | 0.475 | 0.018 | 3.9×10^-08^ | 0.0033 | 231166 | 0.0001616 | 29.7520661 |
| rs710841 | chr 4:82149831 | T | C | 0.2417 | 0.029 | 8.5×10^-14^ | 0.0038 | 230174 | 0.00030828 | 58.2409972 |
| rs7430034 | chr 3:134342127 | T | C | 0.5917 | 0.021 | 3.8×10^-09^ | 0.0035 | 231192 | 0.00021308 | 36 |
| rs757608 | chr 17:59497277 | G | A | 0.7 | -0.027 | 1.0×10^-13^ | 0.0036 | 229039 | 0.00030618 | 56.25 |
| rs7801581 | chr 7:27223771 | T | C | 0.2583 | 0.027 | 8.0×10^-11^ | 0.0042 | 216463 | 0.00027933 | 41.3265306 |
| rs780159 | chr 10:80907147 | G | A | 0.525 | 0.021 | 1.8×10^-09^ | 0.0035 | 220810 | 0.00021995 | 36 |
| rs7970350 | chr 12:66360164 | T | C | 0.4917 | -0.019 | 3.8×10^-08^ | 0.0034 | 229815 | 0.00018045 | 31.2283737 |
| rs798489 | chr 7:2801803 | T | C | 0.275 | -0.025 | 1.3×10^-11^ | 0.0037 | 230932 | 0.00024922 | 45.6537619 |
| rs822531 | chr 7:148629759 | T | C | 0.7333 | 0.024 | 3.7×10^-08^ | 0.0044 | 226664 | 0.0002253 | 29.7520661 |
| rs9389986 | chr 6:142661114 | A | T | 0.25 | -0.024 | 5.7×10^-11^ | 0.0037 | 230700 | 0.000216 | 42.0745069 |
| rs9435732 | chr 1:17308158 | T | C | 0.175 | -0.031 | 4.1×10^-16^ | 0.0038 | 228579 | 0.00027749 | 66.5512465 |
| rs979012 | chr 20:6623374 | C | T | 0.6417 | -0.033 | 5.4×10^-20^ | 0.0036 | 229815 | 0.00050077 | 84.0277778 |
| rs9864077 | chr 3:64704891 | C | T | 0.2417 | -0.022 | 1.3×10^-09^ | 0.0037 | 219478 | 0.00017742 | 35.3542732 |
| rs991967 | chr 1:218615451 | C | A | 0.1897 | 0.026 | 1.1×10^-12^ | 0.0037 | 230157 | 0.00020782 | 49.3791088 |
| rs9977276 | chr 21:47436327 | G | T | 0.75 | 0.022 | 4.4×10^-08^ | 0.004 | 229565 | 0.0001815 | 30.25 |

SNP, single nucleotide polymorphism; SE, standard error

Table S2. 30 SNPs represented genetically predicted WRHadjBMI.

| **SNP** | **Postion** | **Effect_allele** | **Other_allele** | **Effect allele frequency** | **β** | ***P*-value** | **SE** | **Samplesize** | **R2** | **F** |
| --- | --- | --- | --- | --- | --- | --- | --- | --- | --- | --- |
| rs10245353 | chr 7:25858614 | A | C | 0.1833 | 0.035 | 8.4×10^-16^ | 0.0043 | 210008 | 0.00036677 | 66.2520281 |
| rs10804591 | chr 3:129334233 | A | C | 0.85 | 0.024 | 6.6×10^-09^ | 0.0042 | 209921 | 0.00014688 | 32.6530612 |
| rs10842707 | chr 12:26471364 | T | C | 0.1667 | 0.032 | 4.4×10^-16^ | 0.004 | 210023 | 0.00028449 | 64 |
| rs10991437 | chr 9:107735920 | A | C | 0.1 | 0.031 | 1.0×10^-08^ | 0.0054 | 209941 | 0.00017298 | 32.9561043 |
| rs11231693 | chr 11:63862612 | A | G | 0.0417 | 0.041 | 4.5×10^-08^ | 0.0075 | 198072 | 0.00013435 | 29.8844444 |
| rs12143789 | chr 1:119497154 | C | G | 0.1583 | 0.024 | 7.6×10^-09^ | 0.0042 | 209874 | 0.00015349 | 32.6530612 |
| rs12608504 | chr 19:18389135 | G | A | 0.6583 | -0.022 | 8.8×10^-10^ | 0.0036 | 209990 | 0.00021774 | 37.345679 |
| rs12679556 | chr 8:72514228 | G | T | 0.2083 | 0.027 | 2.1×10^-11^ | 0.004 | 203826 | 0.00024044 | 45.5625 |
| rs1294410 | chr 6:6738752 | C | T | 0.625 | 0.031 | 2.0×10^-18^ | 0.0035 | 209830 | 0.00045047 | 78.4489796 |
| rs1385167 | chr 2:66200648 | G | A | 0.1417 | 0.029 | 1.8×10^-09^ | 0.0049 | 206619 | 0.00020457 | 35.0270721 |
| rs1440372 | chr 15:67033151 | C | T | 0.7417 | 0.024 | 1.1×10^-10^ | 0.0038 | 207447 | 0.0002207 | 39.8891967 |
| rs1569135 | chr 2:188115398 | G | A | 0.4667 | -0.021 | 5.6×10^-10^ | 0.0034 | 209906 | 0.00021952 | 38.1487889 |
| rs17451107 | chr 3:156797609 | C | T | 0.375 | -0.026 | 1.1×10^-12^ | 0.0036 | 207795 | 0.00031688 | 52.1604938 |
| rs17819328 | chr 3:12489342 | G | T | 0.45 | 0.021 | 2.4×10^-09^ | 0.0035 | 208809 | 0.0002183 | 36 |
| rs1936805 | chr 6:127452116 | T | C | 0.55 | 0.042 | 3.6×10^-35^ | 0.0034 | 209859 | 0.00087318 | 152.595156 |
| rs2294239 | chr 22:29449477 | G | A | 0.45 | -0.025 | 7.2×10^-13^ | 0.0035 | 209454 | 0.00030938 | 51.0204082 |
| rs2645294 | chr 1:119574587 | T | C | 0.5345 | 0.031 | 1.7×10^-19^ | 0.0035 | 209808 | 0.00047821 | 78.4489796 |
| rs303084 | chr 4:124066948 | A | G | 0.7833 | 0.023 | 3.9×10^-08^ | 0.0042 | 209941 | 0.00017959 | 29.9886621 |
| rs4081724 | chr 19:33824946 | A | G | 0.15 | -0.035 | 7.4×10^-12^ | 0.0051 | 207418 | 0.00031238 | 47.0972703 |
| rs4646404 | chr 17:17420199 | A | G | 0.375 | -0.027 | 1.4×10^-11^ | 0.0039 | 198196 | 0.00034172 | 47.9289941 |
| rs4765219 | chr 12:124440110 | A | C | 0.375 | -0.028 | 1.6×10^-15^ | 0.0036 | 209807 | 0.0003675 | 60.4938272 |
| rs6090583 | chr 20:45558831 | G | A | 0.5667 | -0.022 | 6.2×10^-11^ | 0.0034 | 209435 | 0.00023769 | 41.8685121 |
| rs6772129 | chr 3:64700425 | G | A | 0.2417 | -0.035 | 3.4×10^-21^ | 0.0037 | 210055 | 0.00044904 | 89.4813733 |
| rs714515 | chr 1:172352990 | A | G | 0.5417 | -0.027 | 4.4×10^-15^ | 0.0034 | 203401 | 0.00036196 | 63.0622837 |
| rs8030605 | chr 15:56504598 | A | G | 0.1583 | 0.03 | 8.8×10^-09^ | 0.0053 | 208374 | 0.00023983 | 32.0398718 |
| rs8042543 | chr 15:31708263 | T | C | 0.15 | -0.026 | 1.2×10^-09^ | 0.0043 | 208255 | 0.00017238 | 36.5603029 |
| rs878639 | chr 20:33894463 | G | A | 0.3583 | -0.021 | 5.1×10^-09^ | 0.0035 | 207490 | 0.00020279 | 36 |
| rs905938 | chr 1:154991389 | C | T | 0.325 | -0.025 | 7.3×10^-10^ | 0.004 | 207867 | 0.00027422 | 39.0625 |
| rs979012 | chr 20:6623374 | C | T | 0.6417 | -0.027 | 3.3×10^-14^ | 0.0036 | 209941 | 0.00033522 | 56.25 |
| rs9991328 | chr 4:89713121 | T | C | 0.4833 | 0.018 | 4.5×10^-08^ | 0.0034 | 209925 | 0.00016182 | 28.0276817 |

SNP, single nucleotide polymorphism; SE, standard error

**Table S3.** MR analysis of the association between WCadjBMI and LVEDV after removing the outlier

|  |  |  | **IVW** |  |  | **MR Egger** |  |  | **Weighted median** |  |
| --- | --- | --- | --- | --- | --- | --- | --- | --- | --- | --- |
| **Exposure** | **No. of SNPs** | **Outcome** | **β ± SE** | ***P*-value** |  | **β ± SE** | ***P*-value** |  | **β ± SE** | ***P*-value** |
| WCadjBMI | 51 | LVEDV | -0.1718 ± 0.0813 | 0.0345 |  | 0.4372 ± 0.3363 | 0.1996 |  | -0.1807 ± 0.0970 | 0.0625 |

WCadjBMI, waist circumference adjusted for body mass index; SNP, single nucleotide polymorphism; IVW, inverse-variance weighted; SE, standard error; LVEDV, left ventricular end-diastolic volume.

**Table S4**. Heterogeneity and horizontal pleiotropy test of the associations between WCadjBMI and LVEDV after removing the outlier

|  |  |  | **IVW** | |  | **MR-Egger** | | | | |
| --- | --- | --- | --- | --- | --- | --- | --- | --- | --- | --- |
| **Exposure** | **Outcome** |  | **Cochran’s Q** | ***P-value*** |  | **Cochran’s Q** | ***P-value*** | **Intercept** | **SE** | ***P*-value for intercept** |
| WCadjBMI | LVEDV |  | 72.70 | 0.0156 |  | 77.85 | 0.0071 | -0.0160 | 0.0086 | 0.0684 |

WCadjBMI, waist circumference adjusted for body mass index; LVEDV, left ventricular end-diastolic volume; IVW, inverse-variance weighted; SE, standard error.

**Figure S1.** Scatterplots of the association between WCadjBMI and LV parameters.


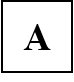

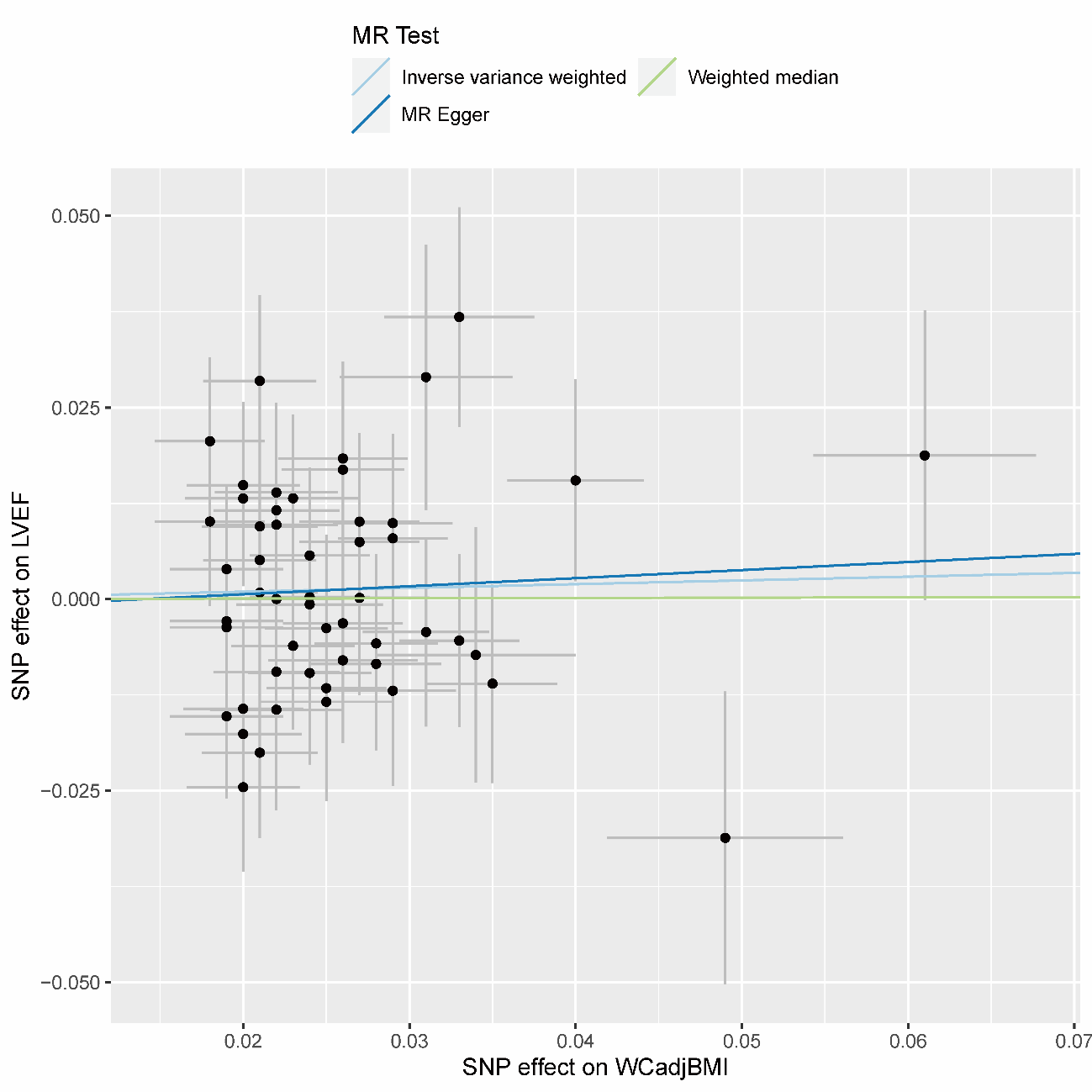


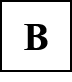

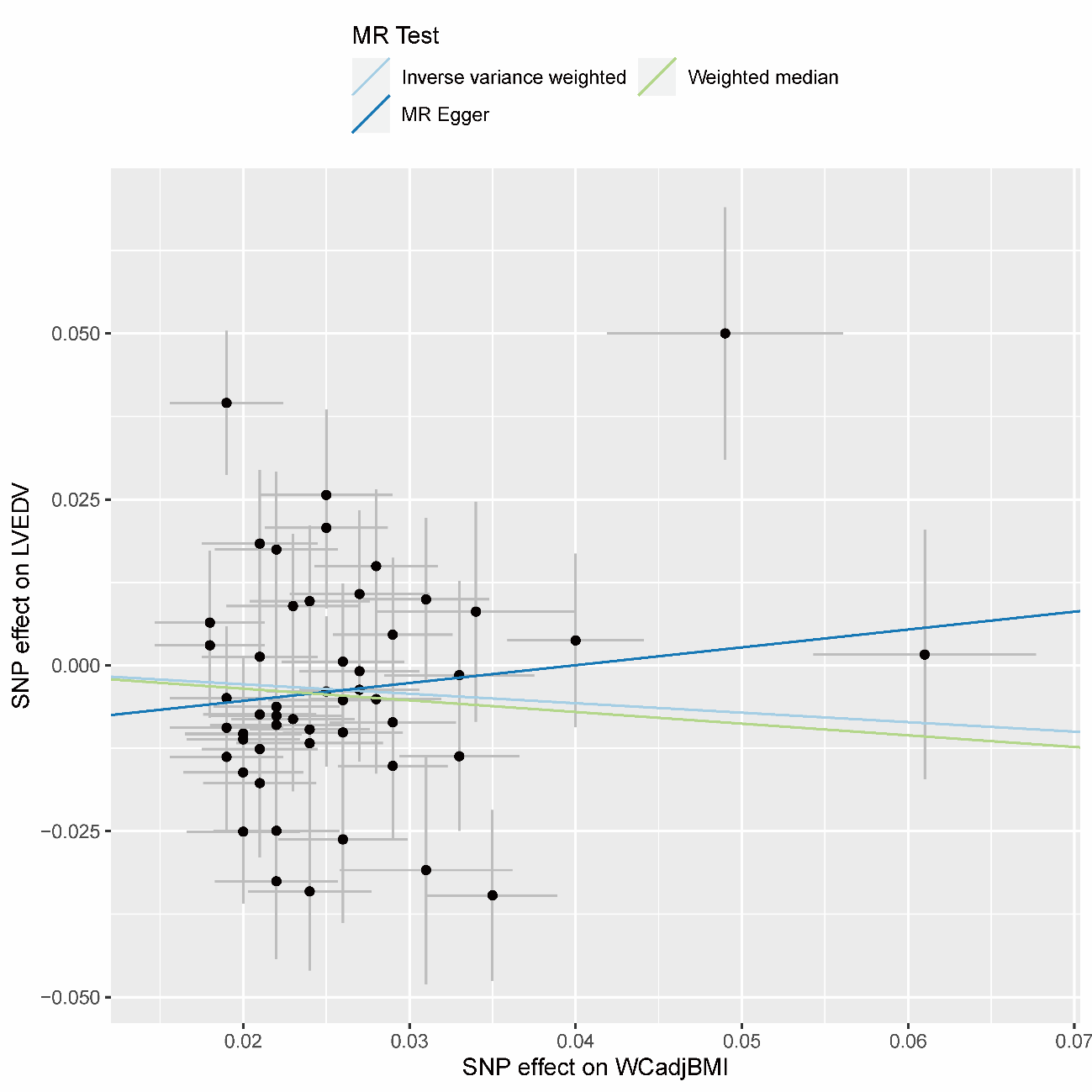


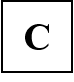

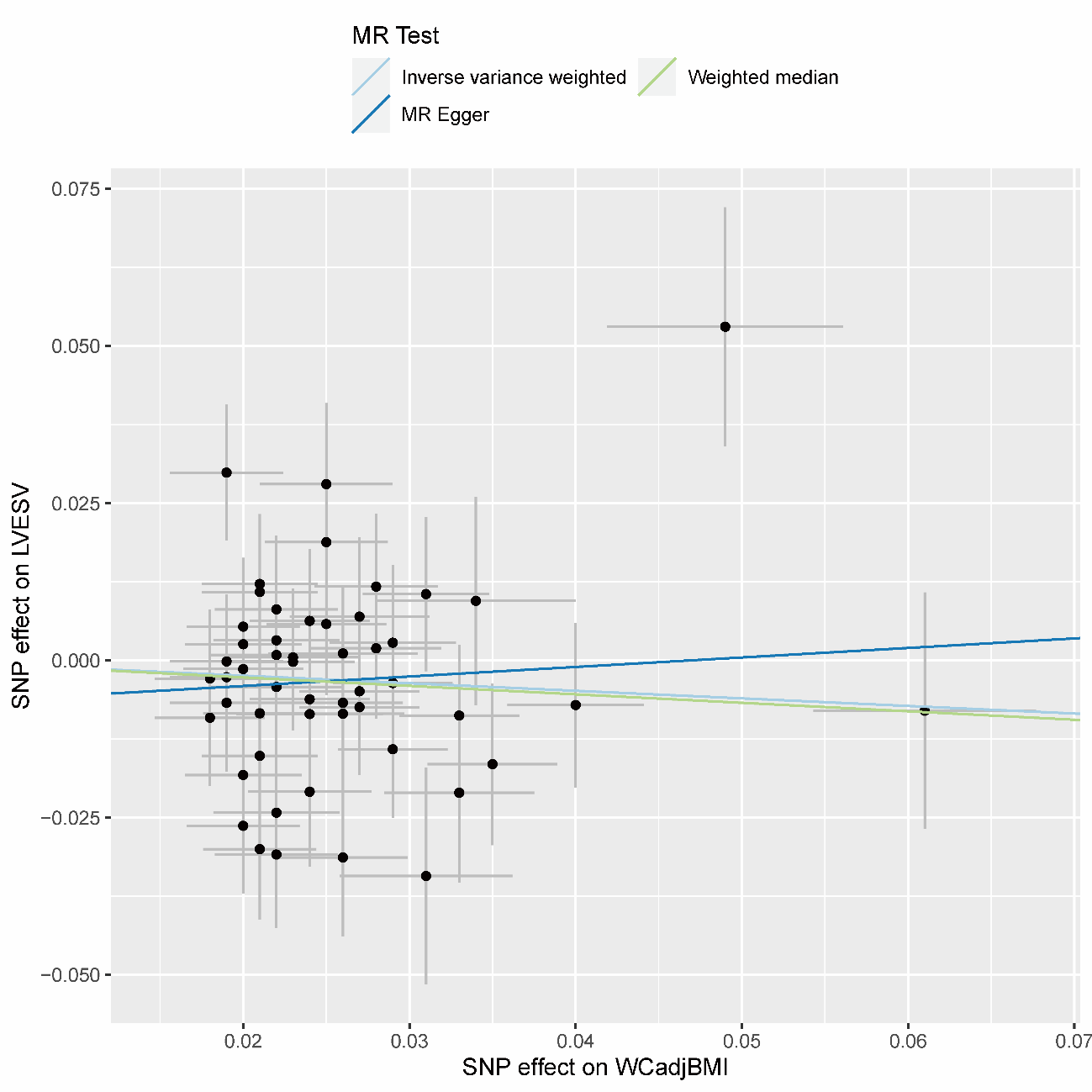


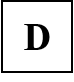

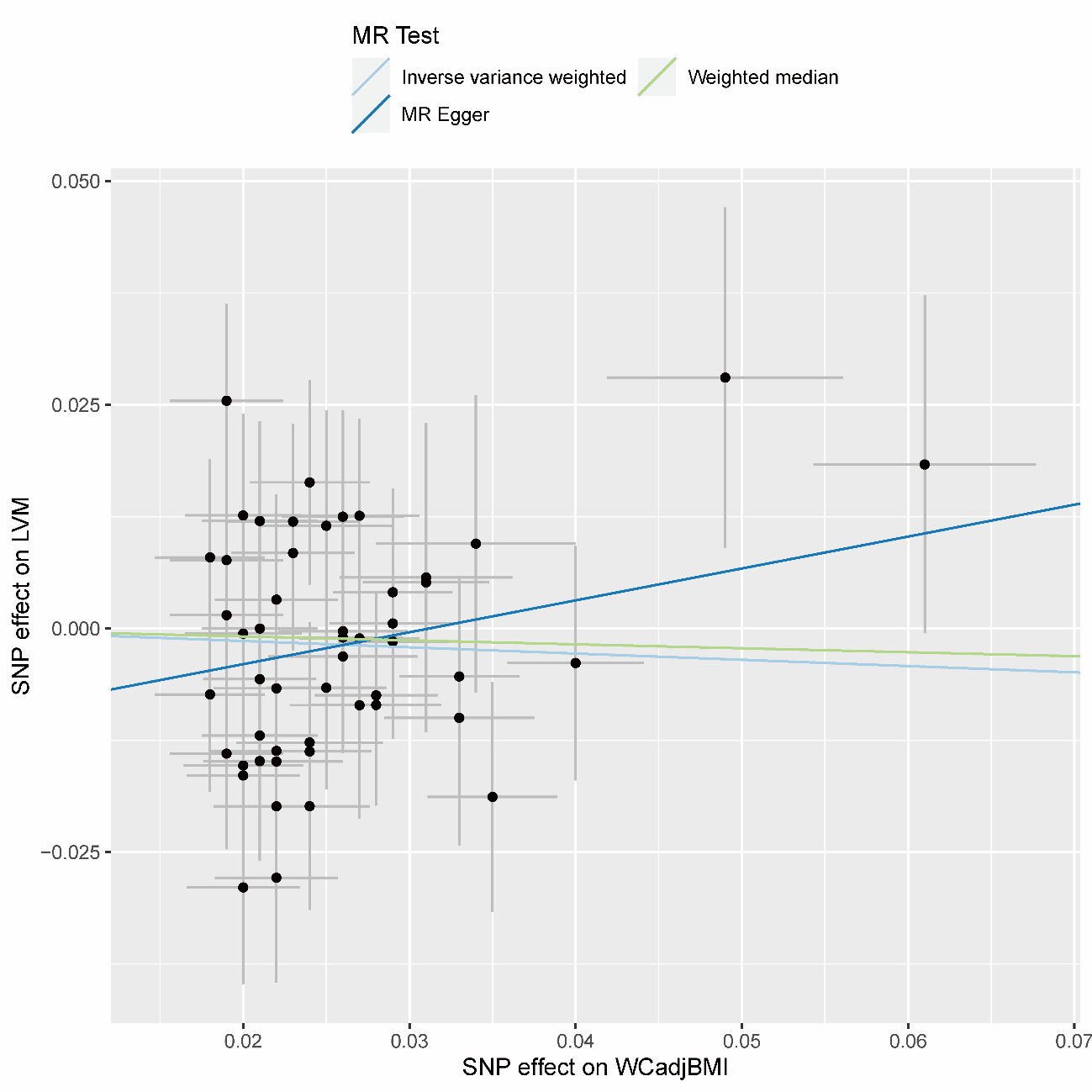


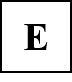

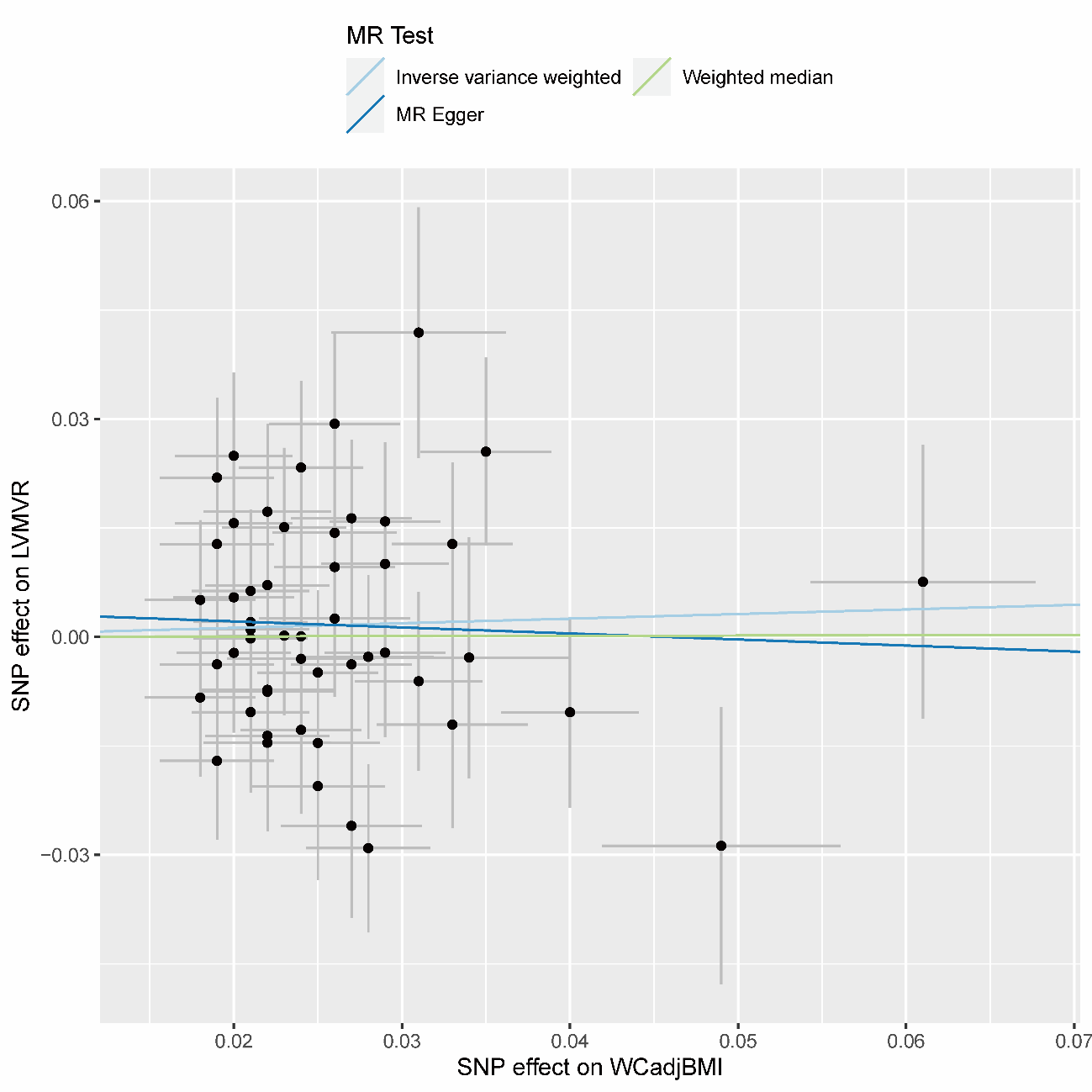


**Figure S1.** Scatterplots of SNP effects on WCadjBMI versus their effects on LVEF (A), LVEDV (B), LVESV (C), LVM (D), LVMVR (E), with the slope of each line corresponding to the estimated MR effect of inverse variance-weighted, Weighted median, and MR-Egger, respectively. Error bars indicate 95% CIs. SNPs, single nucleotide polymorphisms; WCadjBMI, waist circumference adjusted for body mass index; LV, left ventricular; LVEF, LV ejection fraction; LVEDV, LV end-diastolic volume; LVESV, LV end-systolic volume; LVM, LV mass; LVMVR, LV mass-to-end-diastolic volume ratio.

**Figure S2.** Scatterplots of the association between WHRadjBMI and LV parameters.


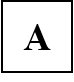

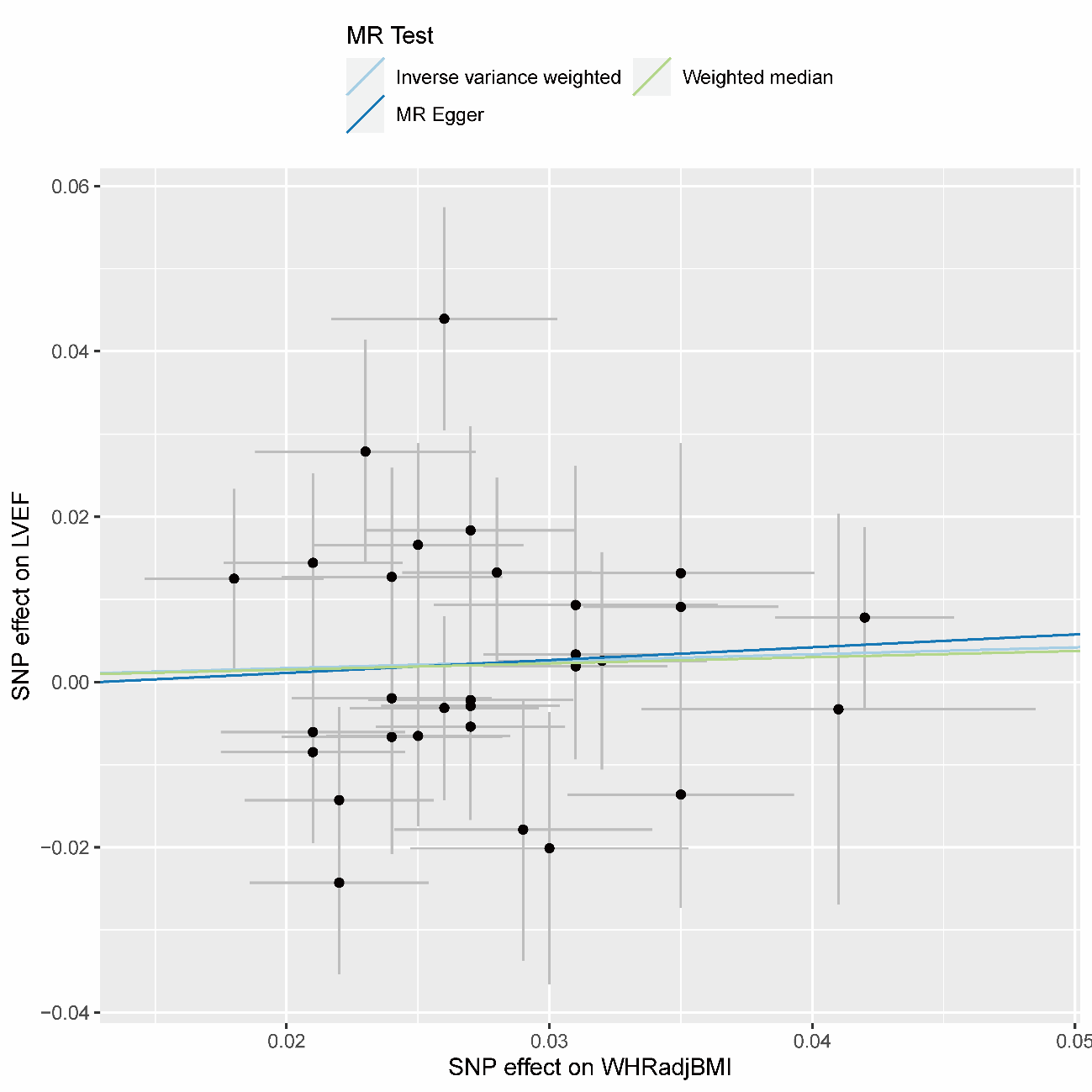


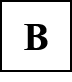

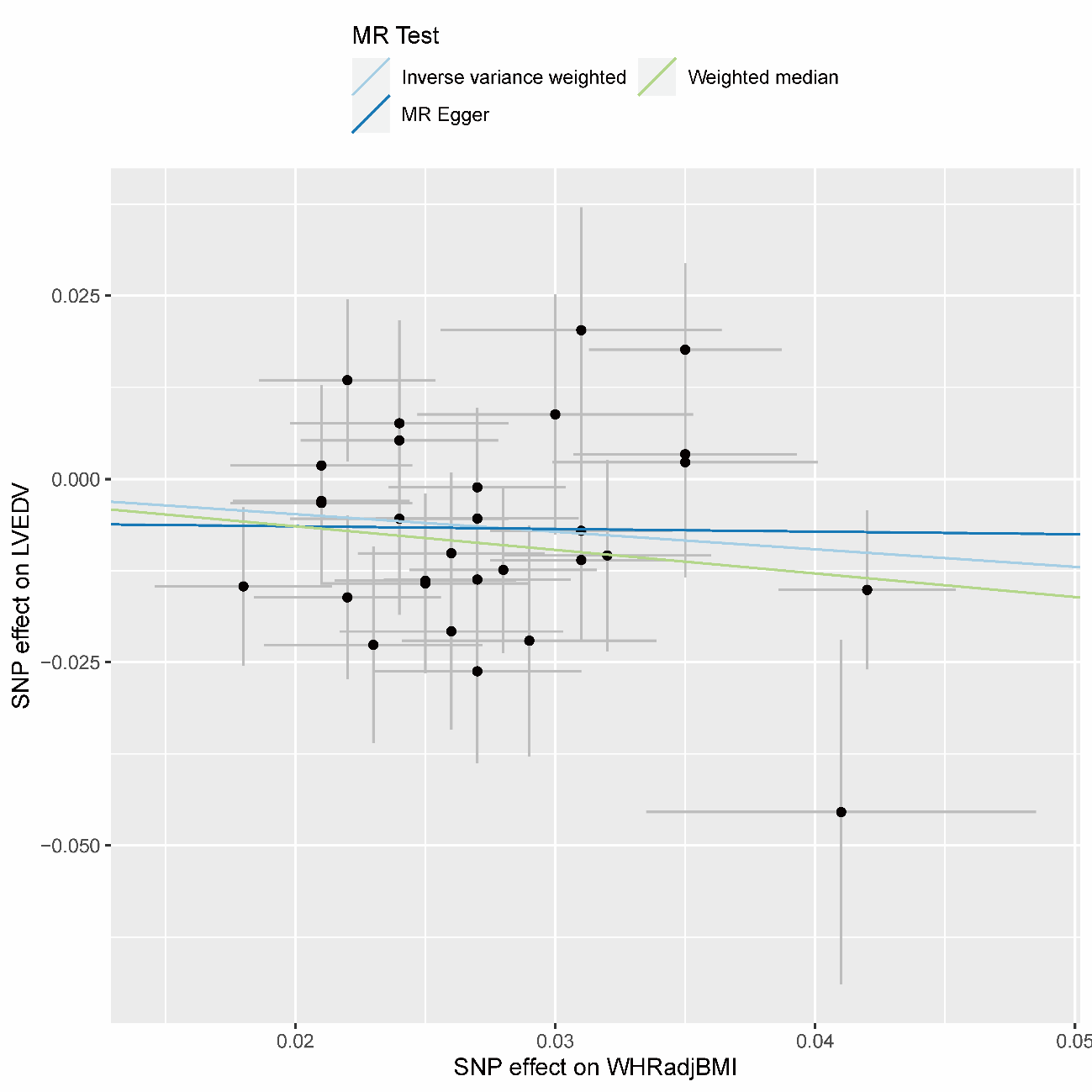


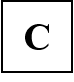

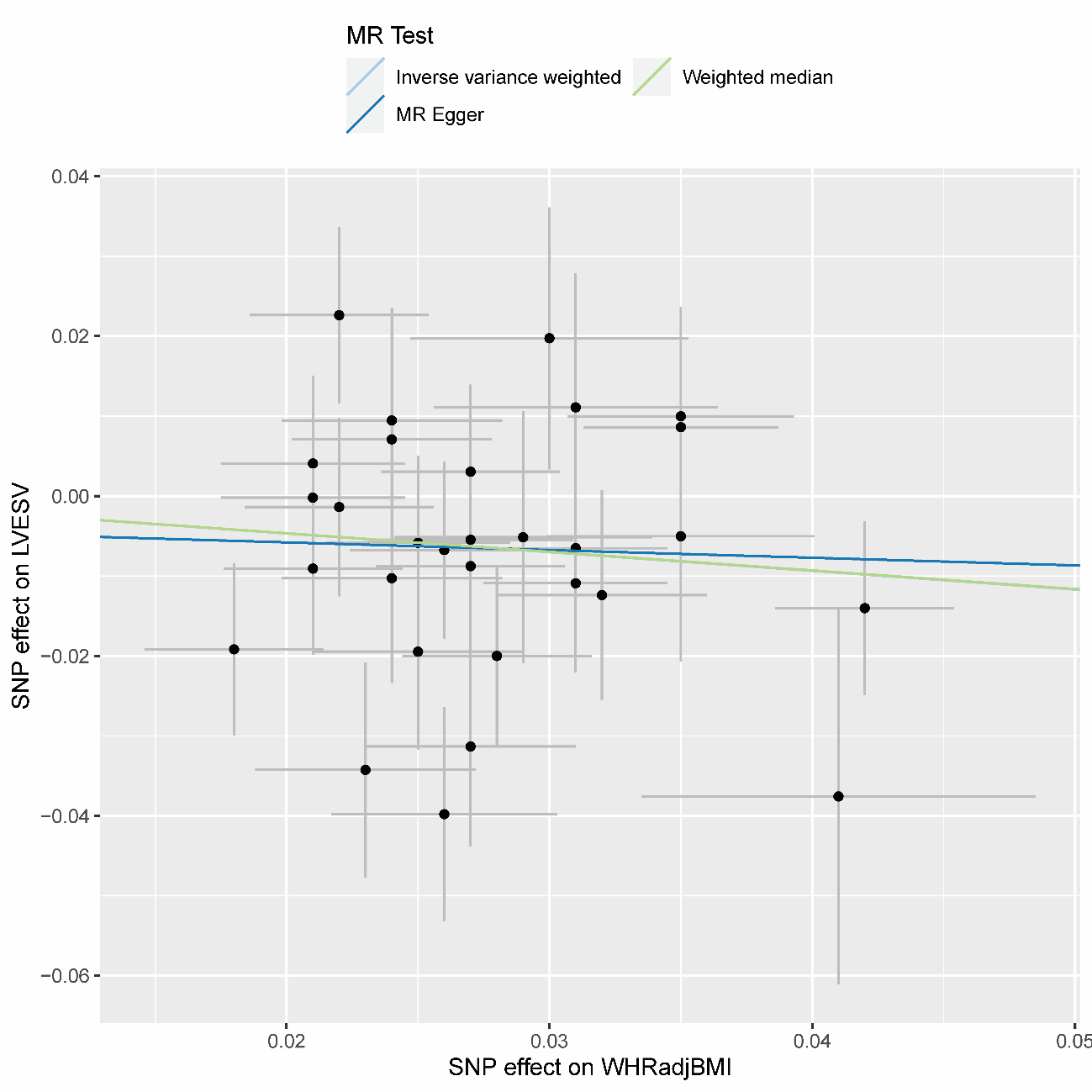


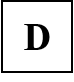

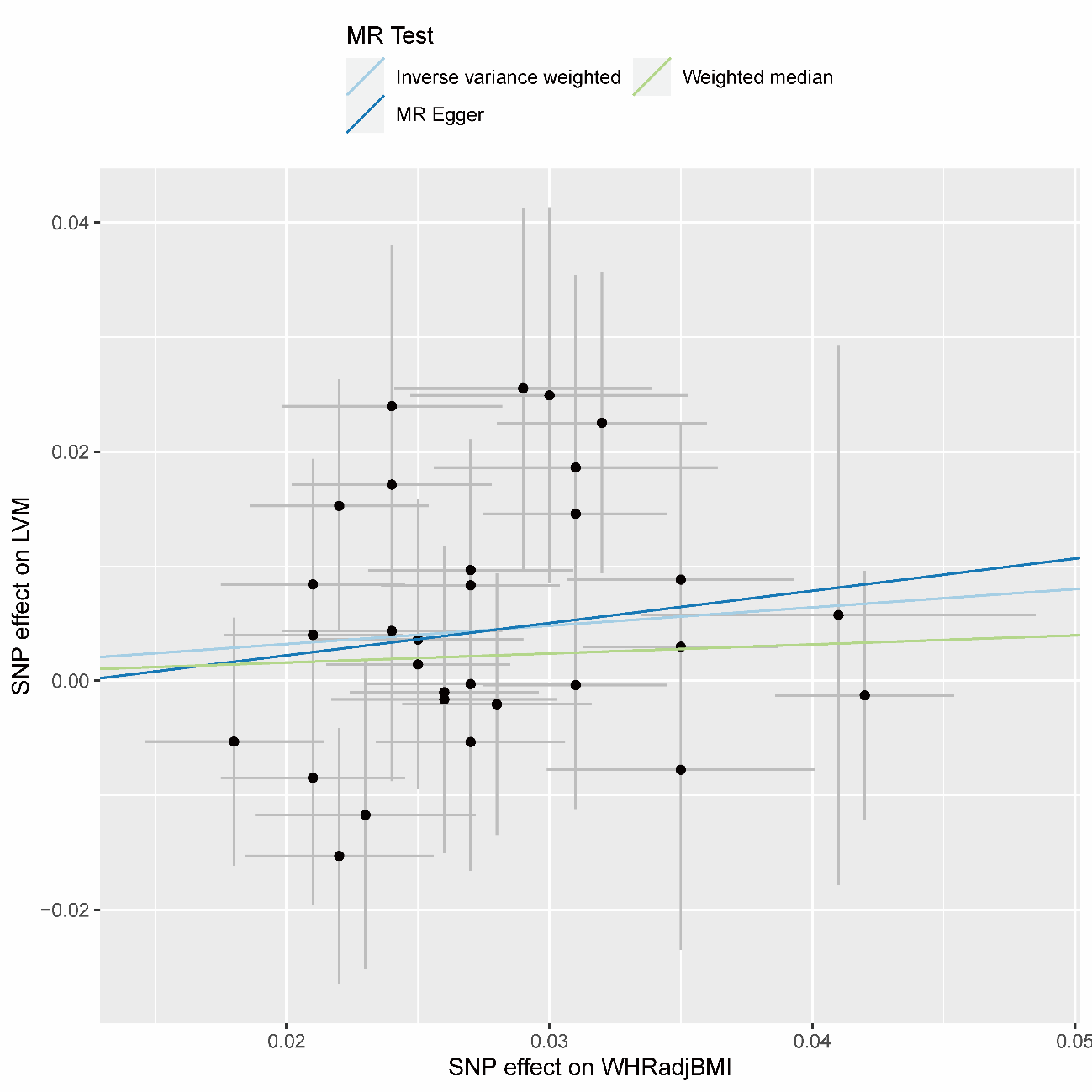


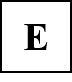

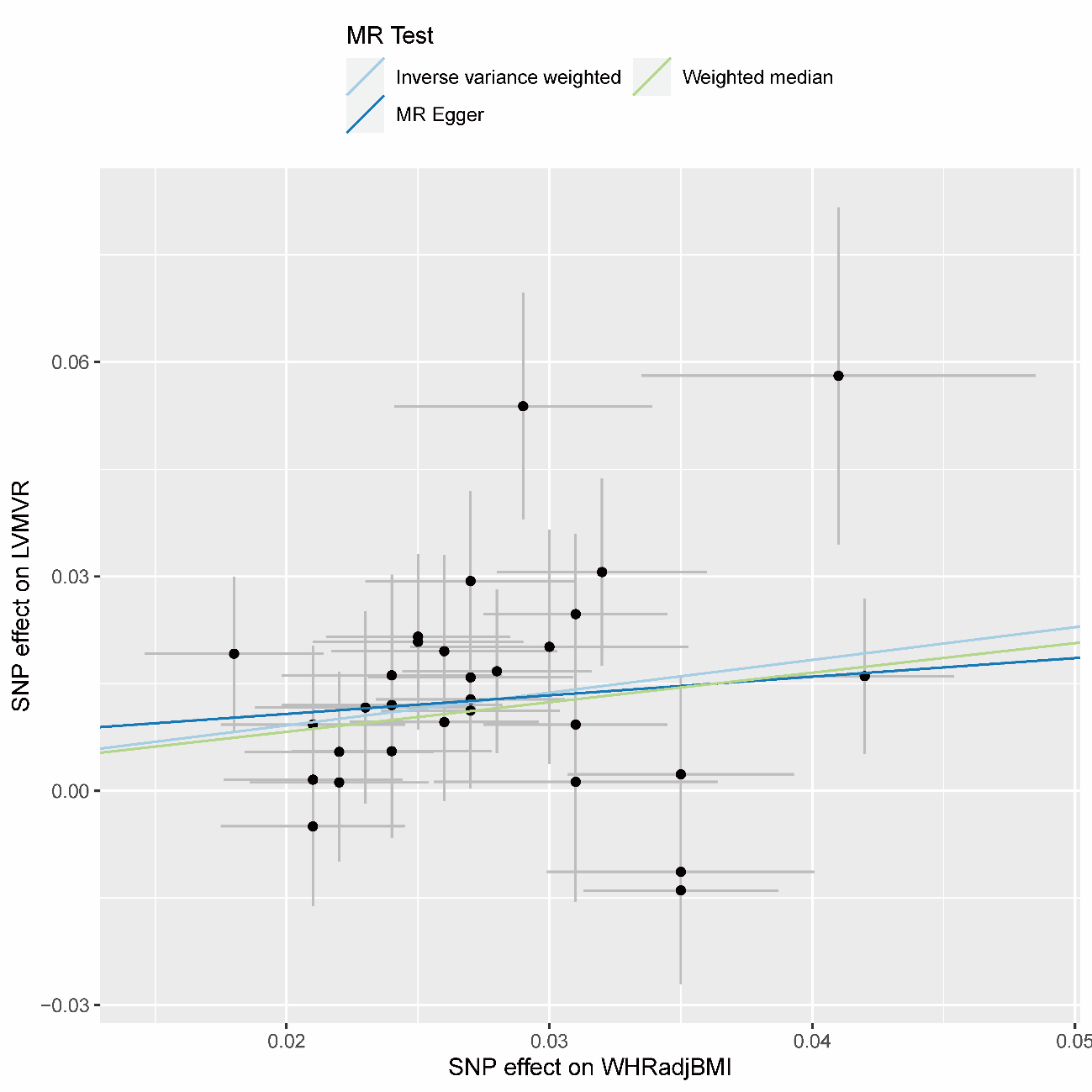


**Figure S2.** Scatterplots of SNP effects on WHRadjBMI versus their effects on LVEF (A), LVEDV (B), LVESV (C), LVM (D), LVMVR (E), with the slope of each line corresponding to the estimated MR effect of inverse variance-weighted, Weighted median, and MR-Egger, respectively. Error bars indicate 95% CIs. SNPs, single nucleotide polymorphisms; WHRadjBMI, waist-to-hip ratio adjusted for body mass index; LV, left ventricular; LVEF, LV ejection fraction; LVEDV, LV end-diastolic volume; LVESV, LV end-systolic volume; LVM, LV mass; LVMVR, LV mass-to-end-diastolic volume ratio.

**Figure S3.** Funnel plots of the association between WCadjBMI and LV parameters.


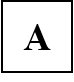

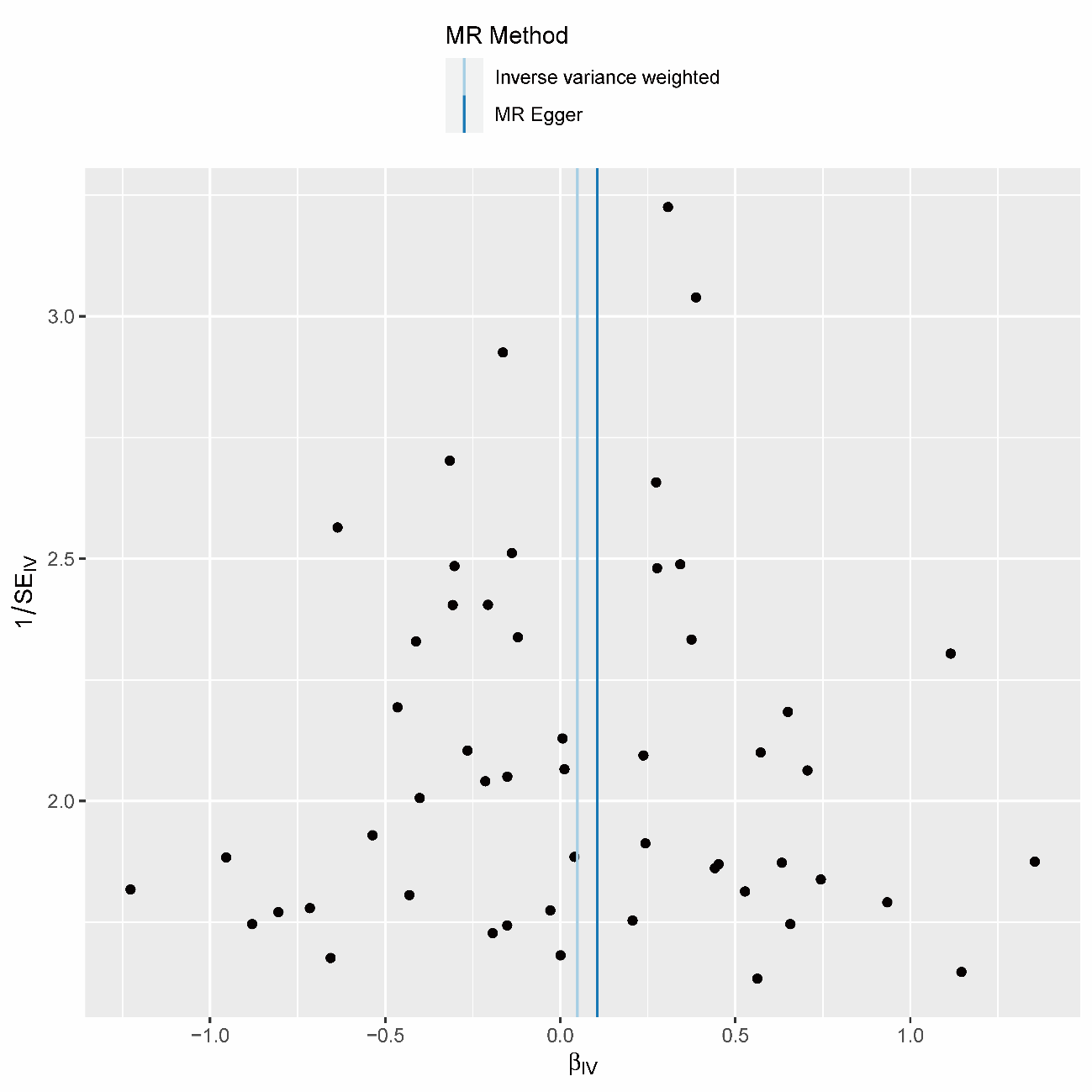


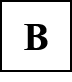

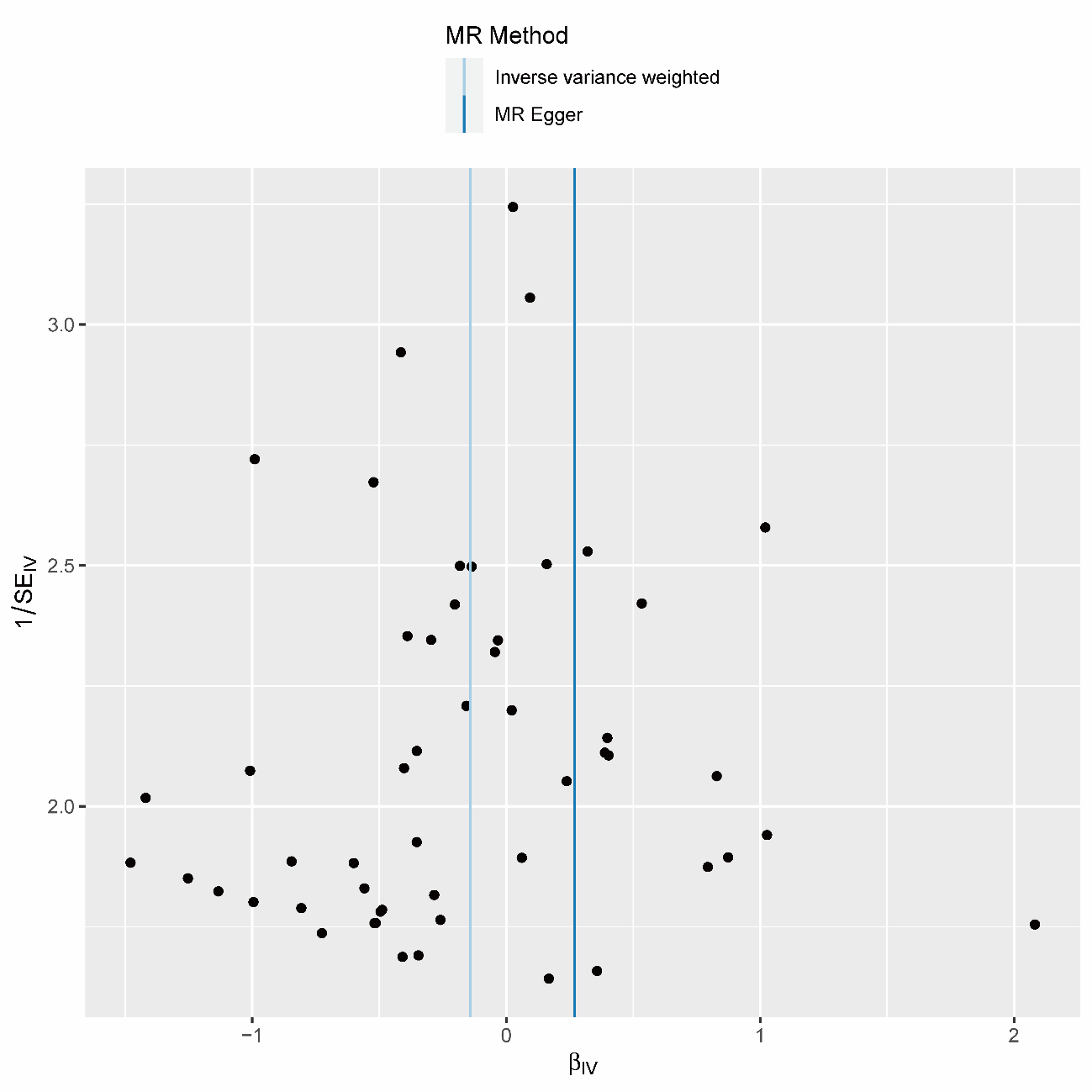


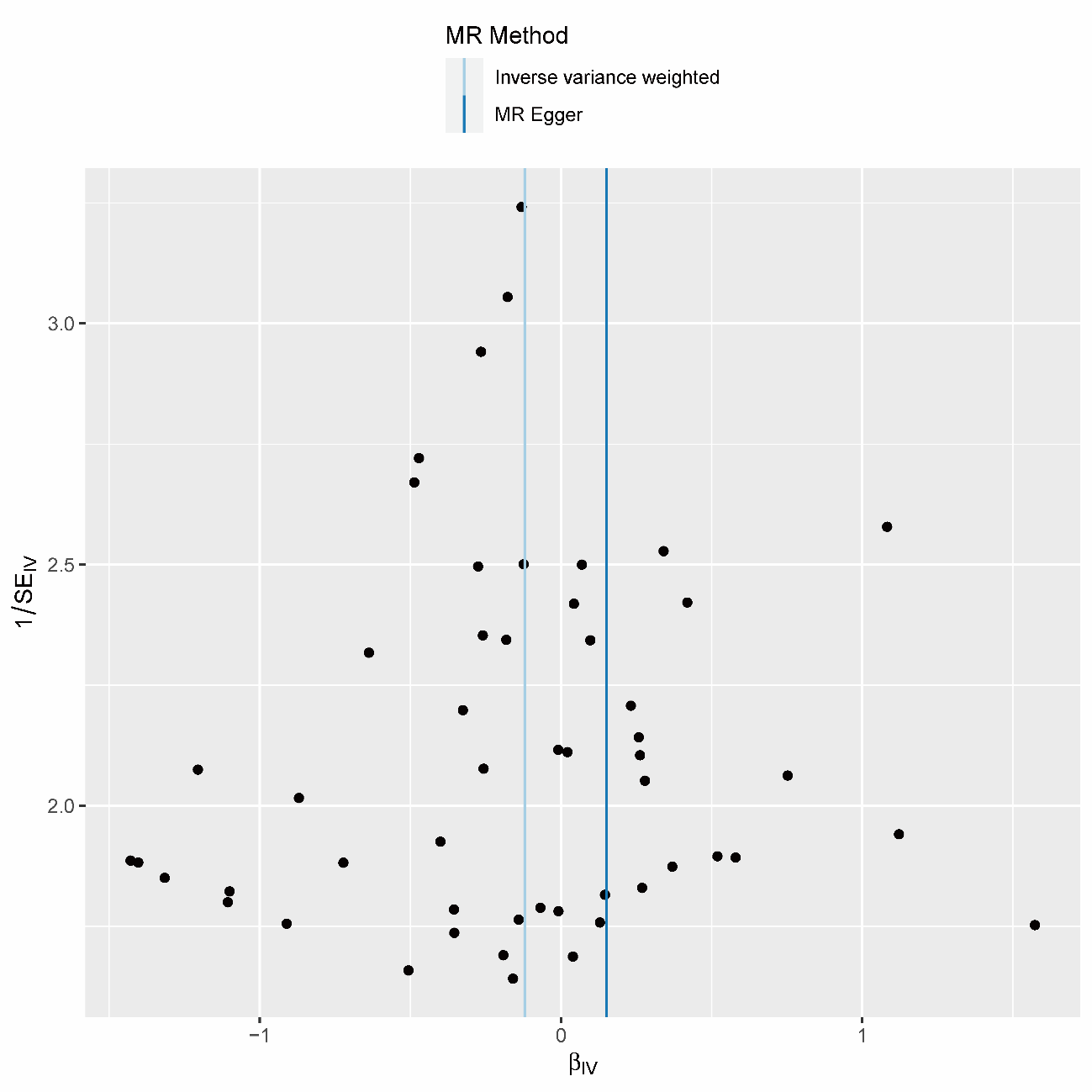

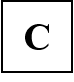


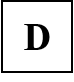

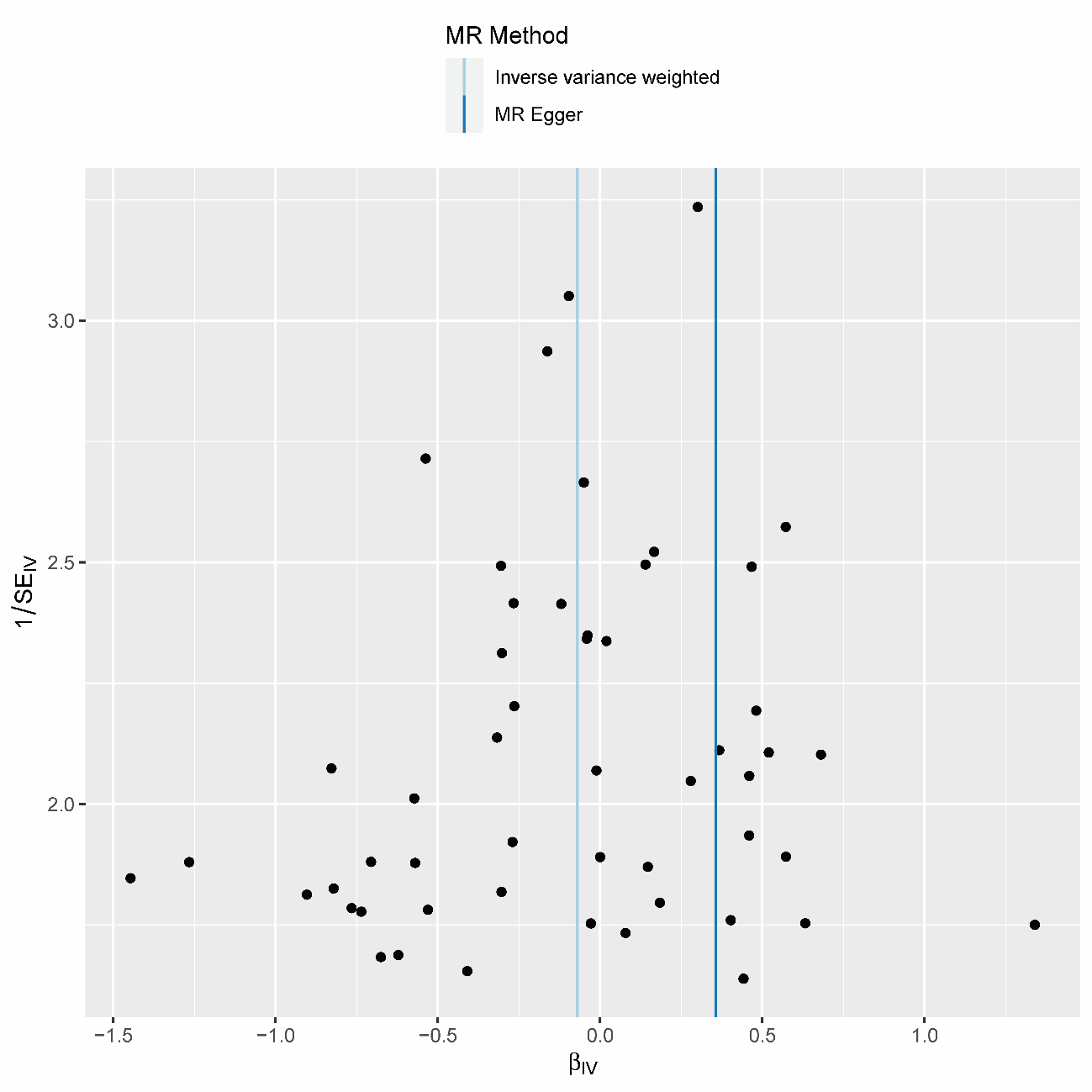


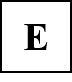

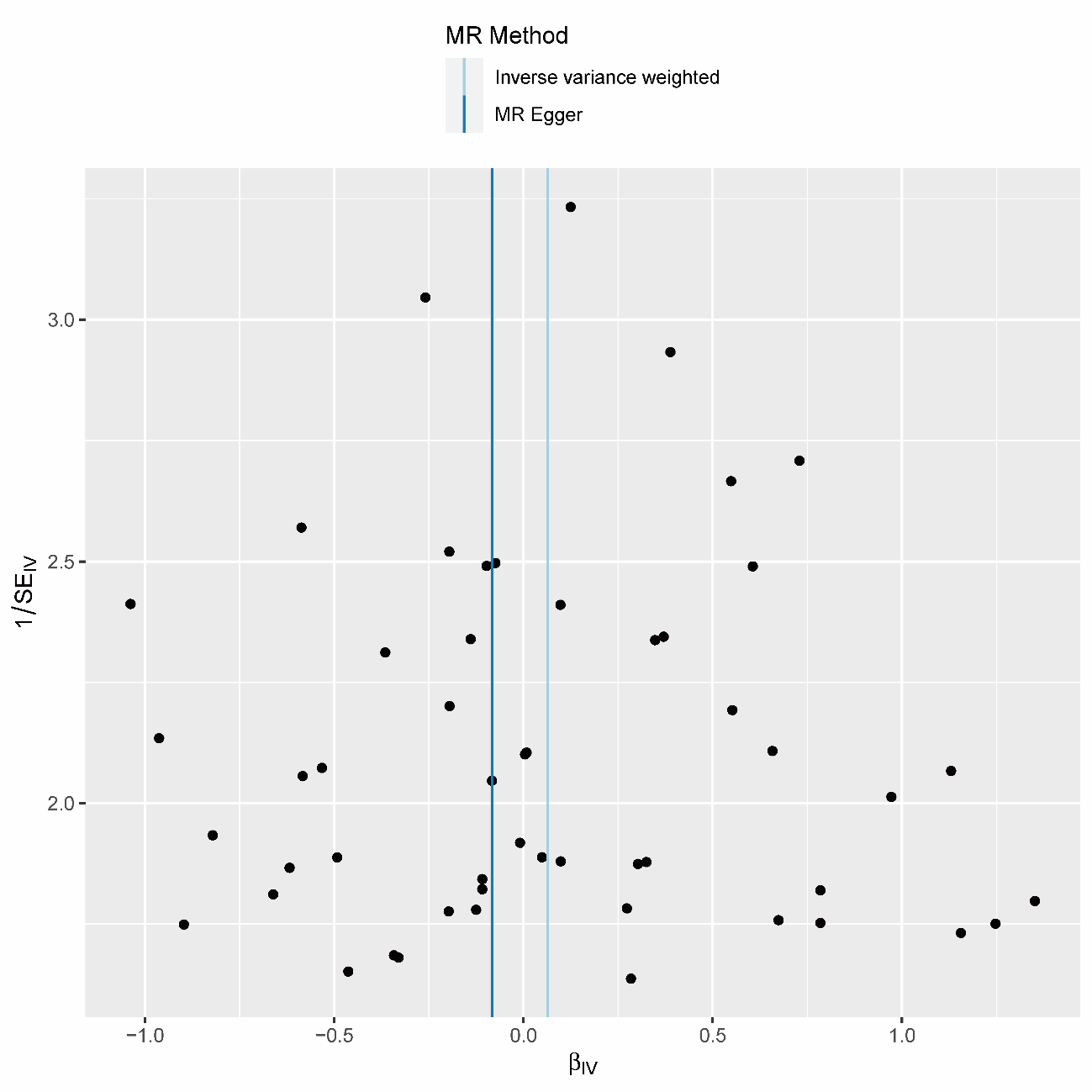


**Figure S3.** Funnel plots of the association between WCadjBMI and LVEF (A), LVEDV (B), LVESV (C), LVM (D), LVMVR (E). WCadjBMI, waist circumference adjusted for body mass index; LV, left ventricular; LVEF, LV ejection fraction; LVEDV, LV end-diastolic volume; LVESV, LV end-systolic volume; LVM, LV mass; LVMVR, LV mass-to-end-diastolic volume ratio.

**Figure S4.** Funnel plots of the association between WHRadjBMI and LV parameters.


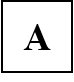

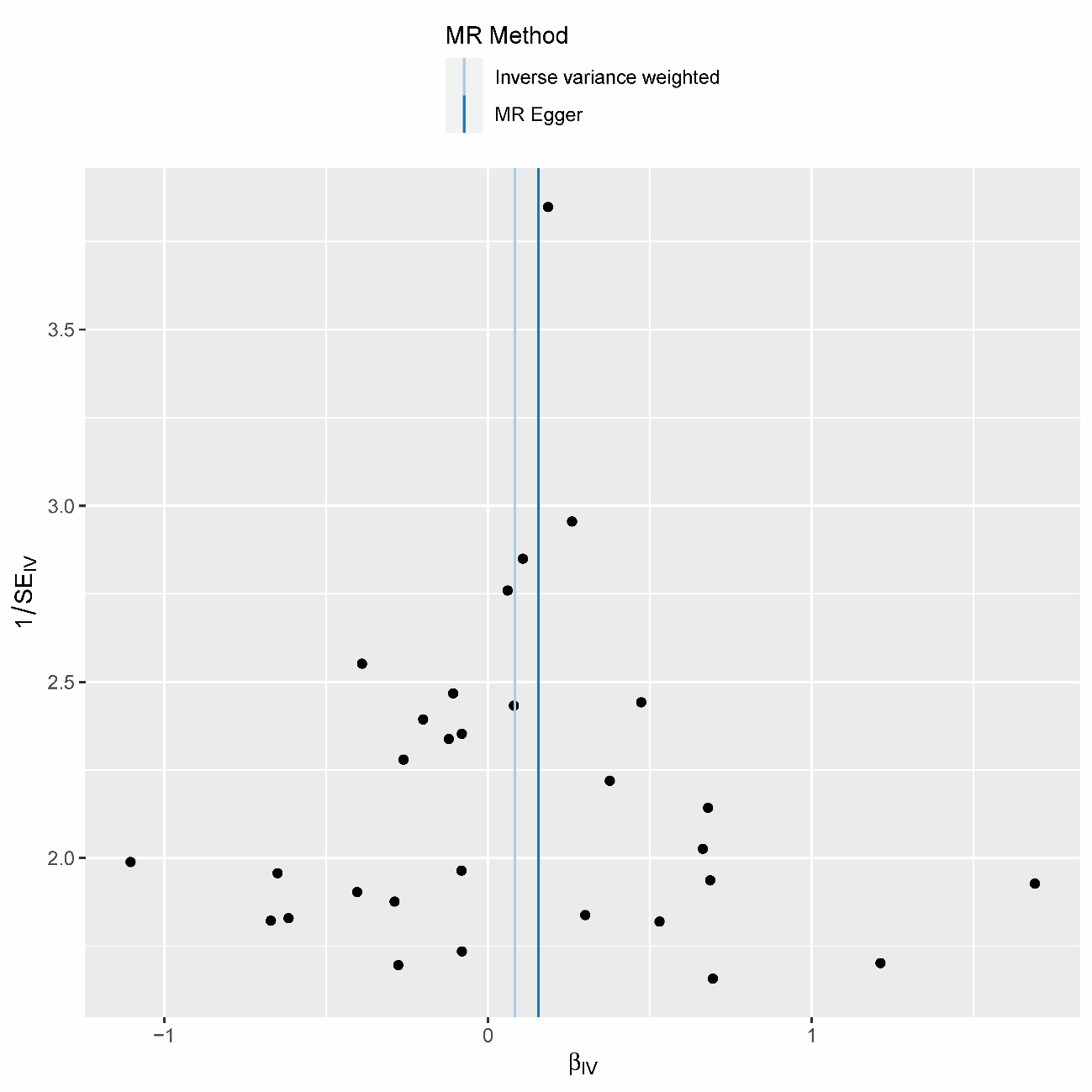


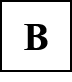

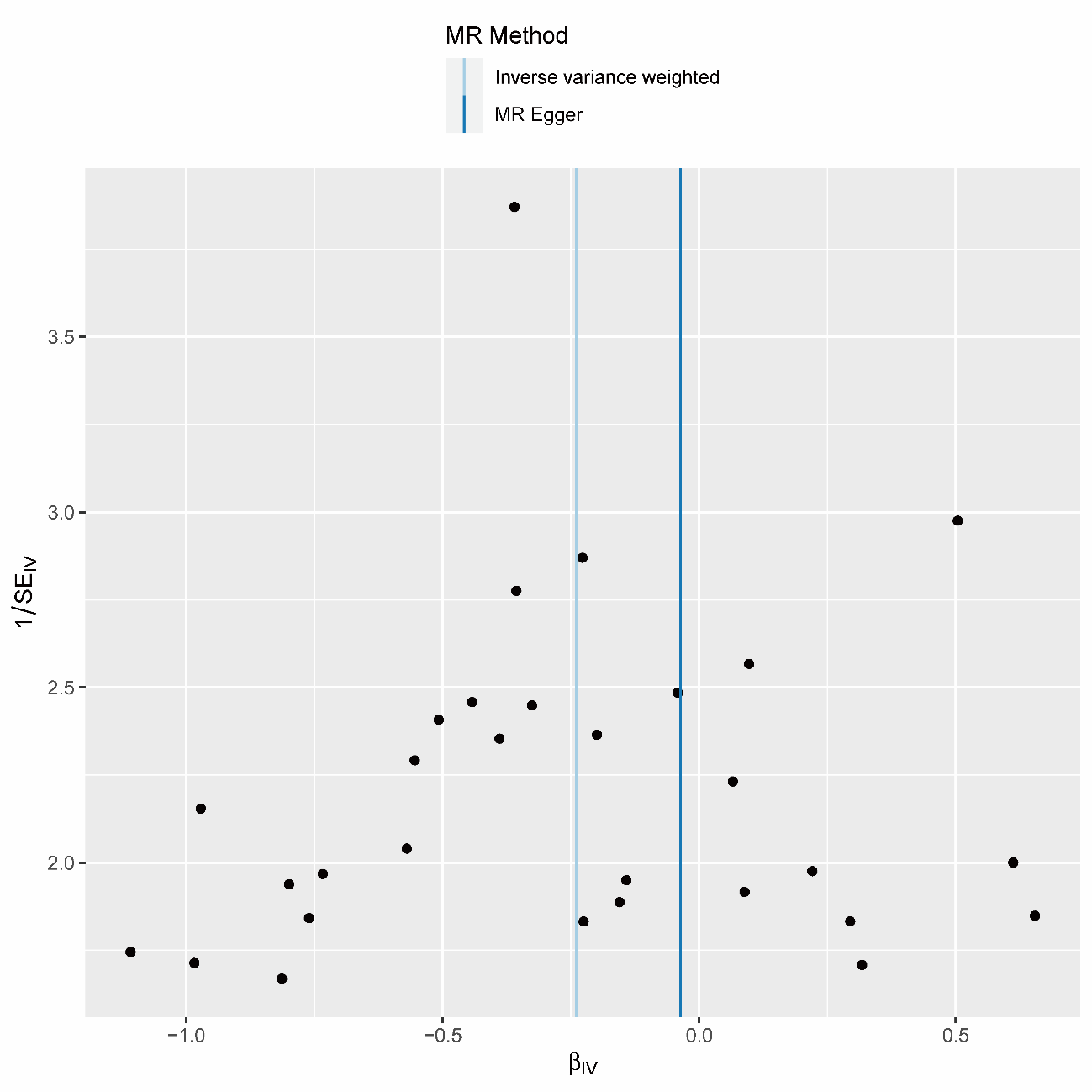


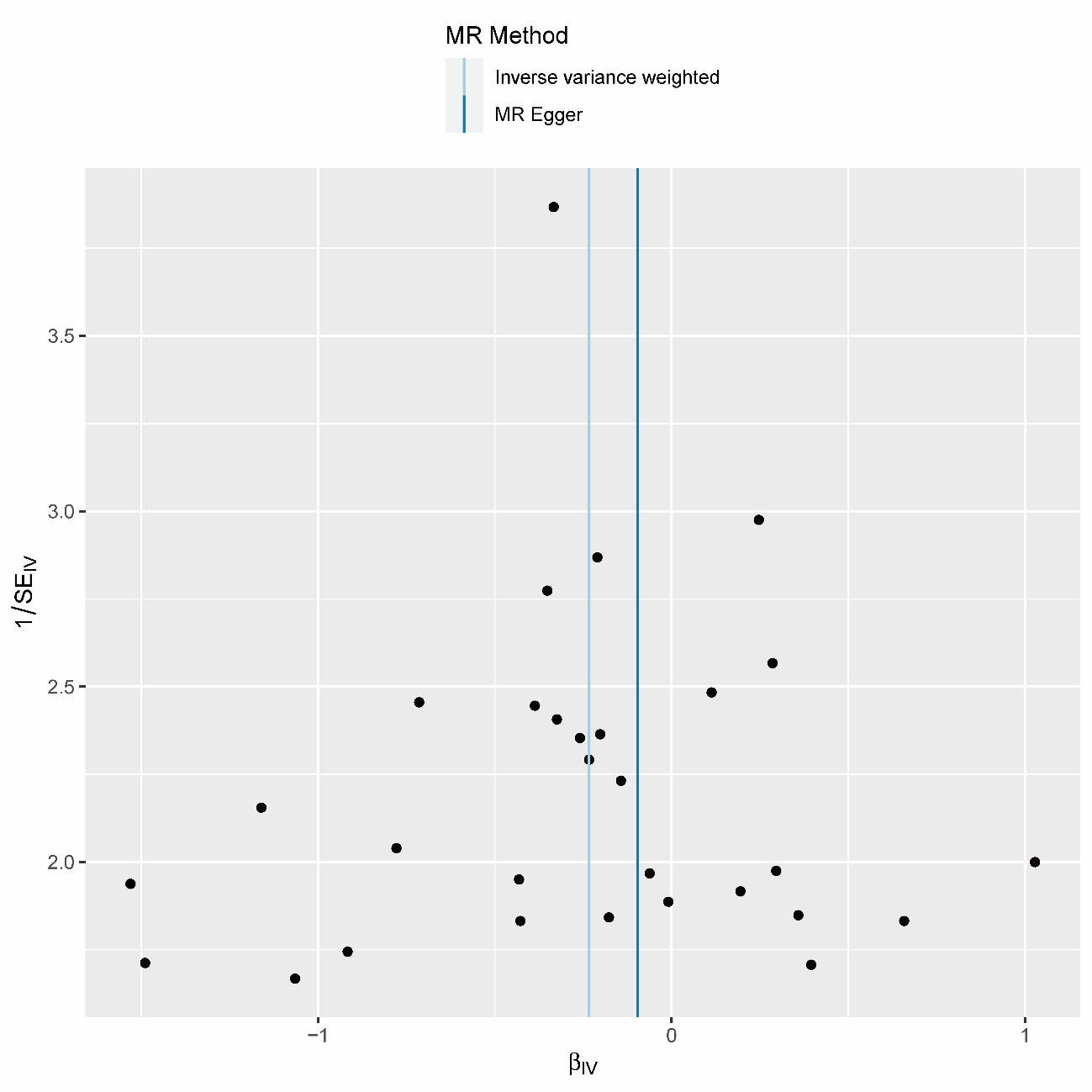

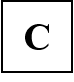

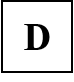


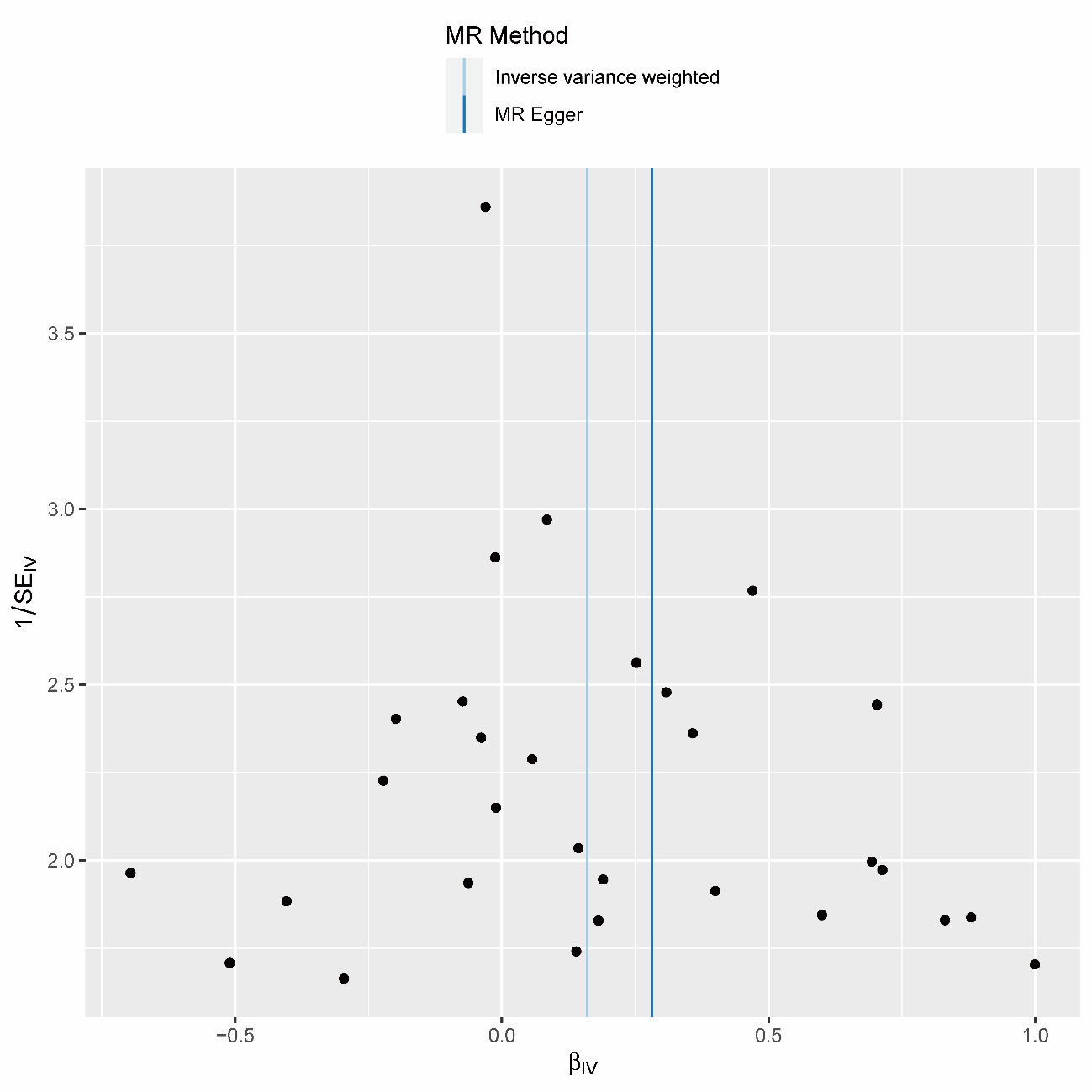


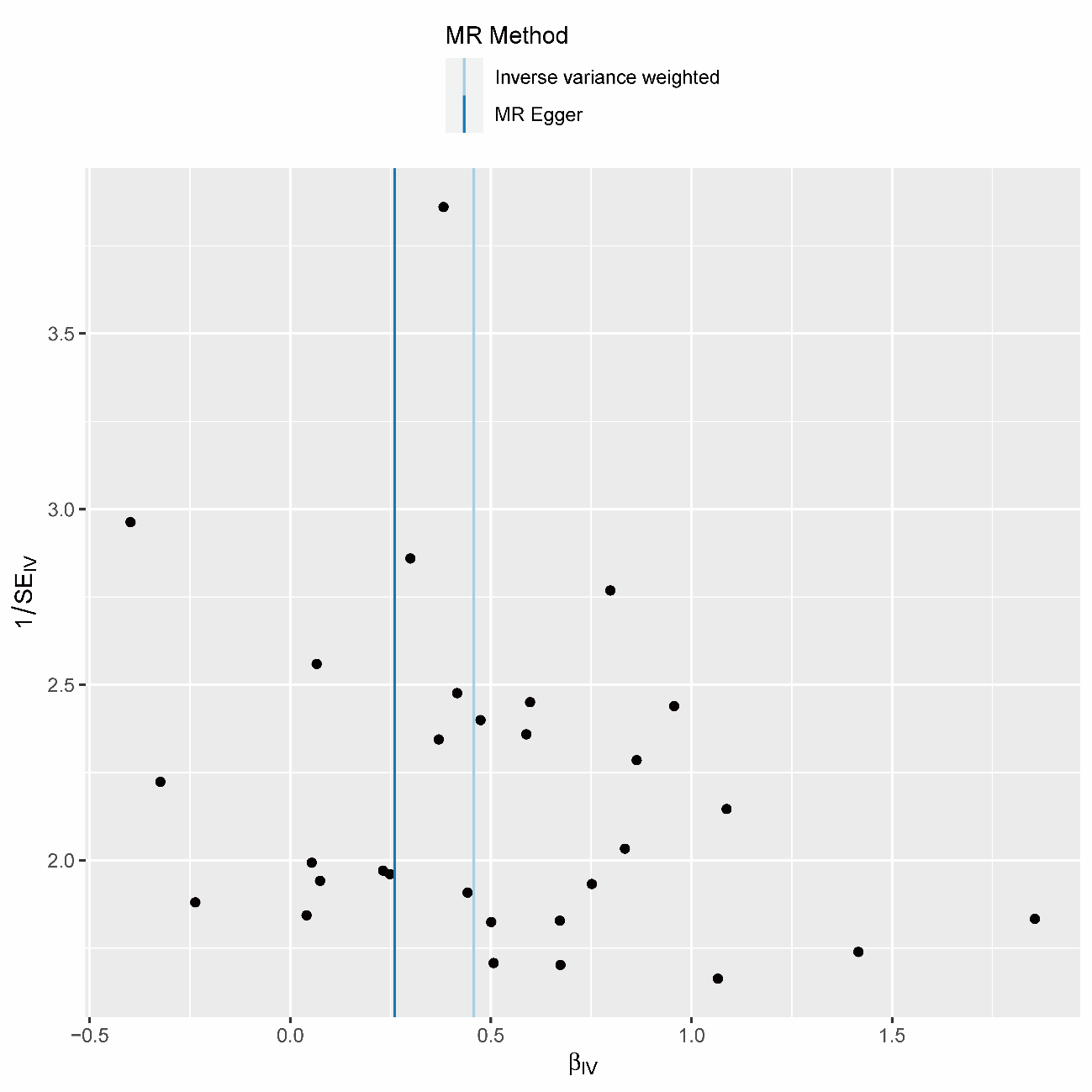

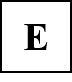


**Figure S4.** Funnel plots of the association between WHRadjBMI and LVEF (A), LVEDV (B), LVESV (C), LVM (D), LVMVR (E). WHRadjBMI, waist-to-hip ratio adjusted for body mass index; LV, left ventricular; LVEF, LV ejection fraction; LVEDV, LV end-diastolic volume; LVESV, LV end-systolic volume; LVM, LV mass; LVMVR, LV mass-to-end-diastolic volume ratio.

**Figure S5.** Leave-one-out analyses of the association between WCadjBMI and LV parameters.


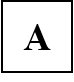


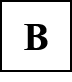


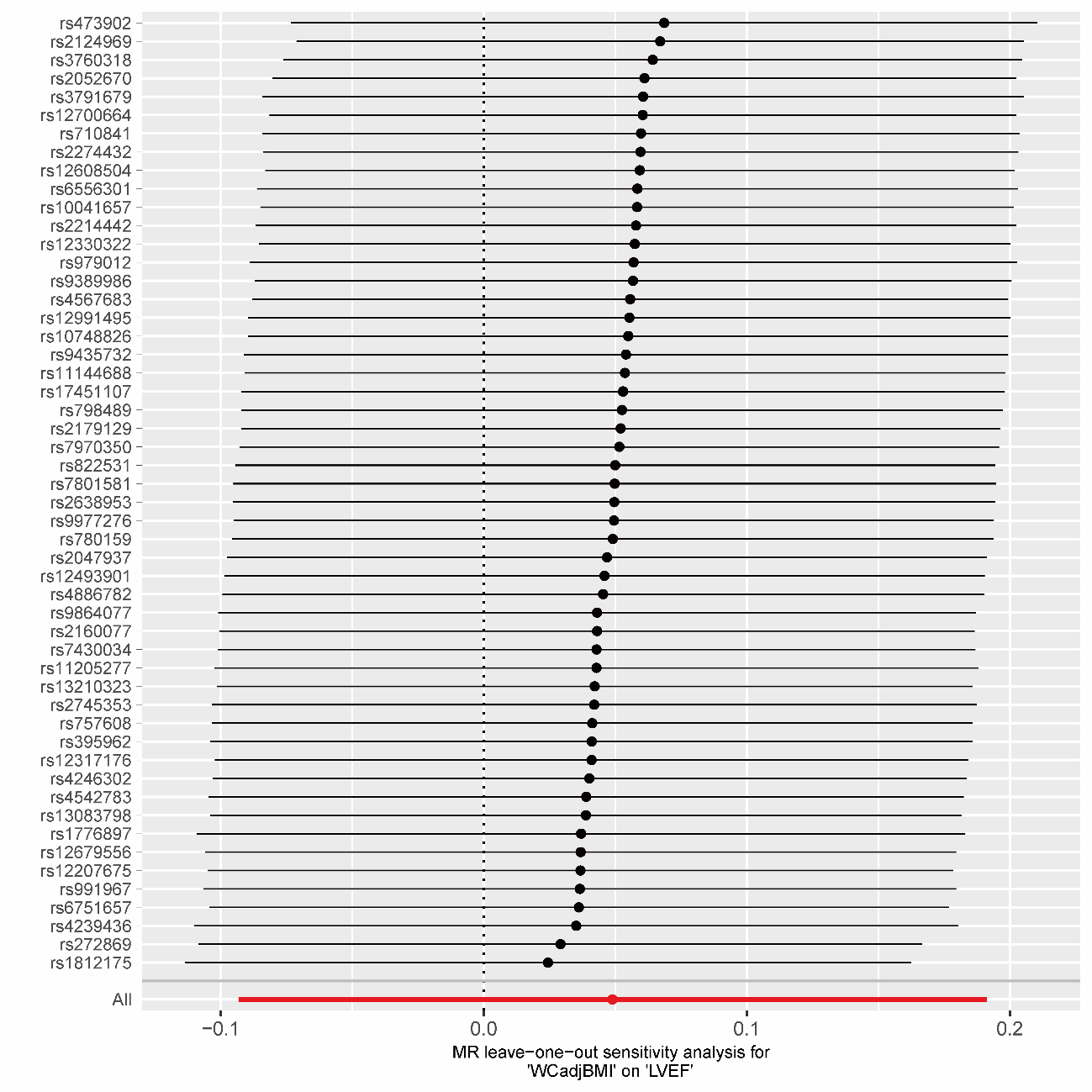


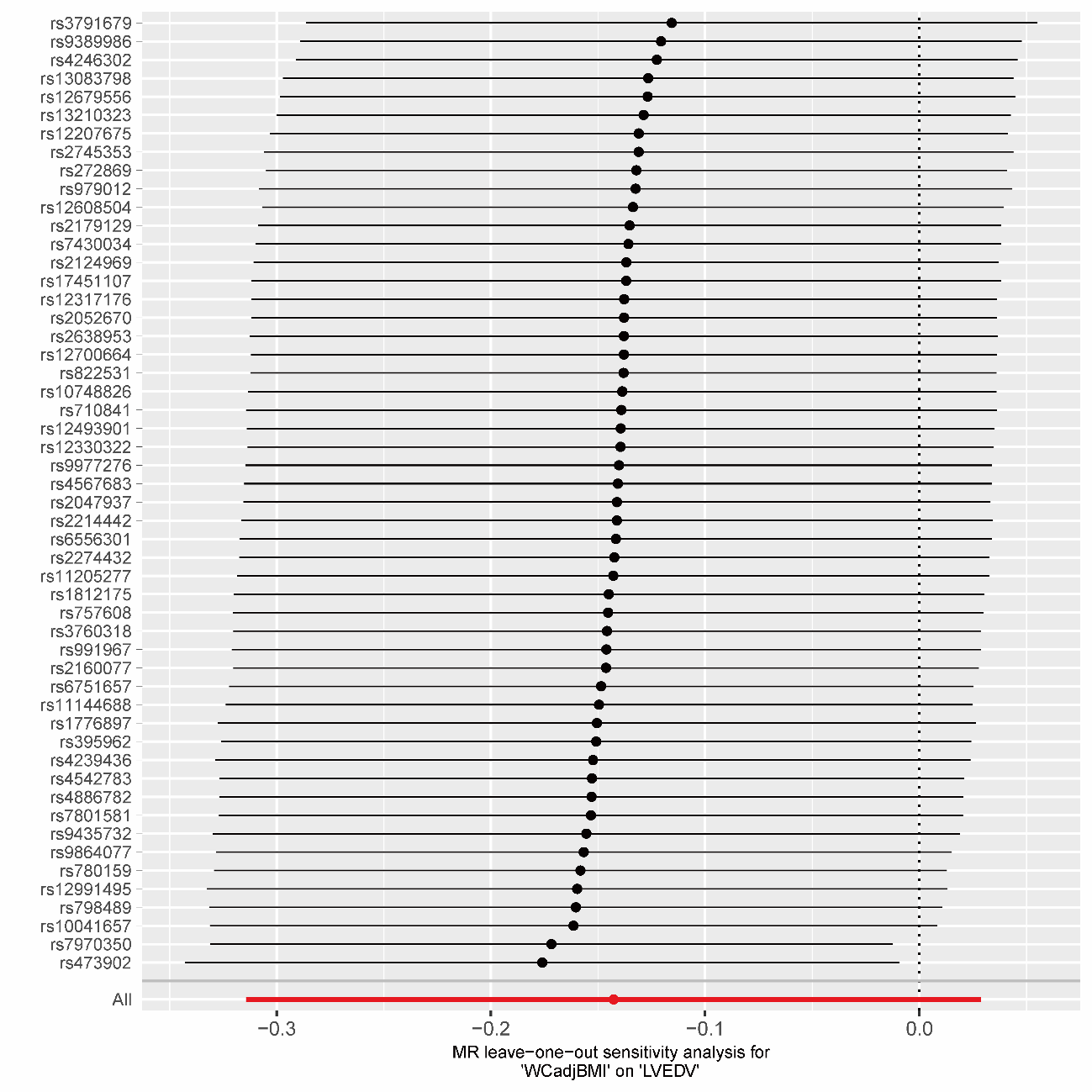


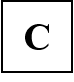


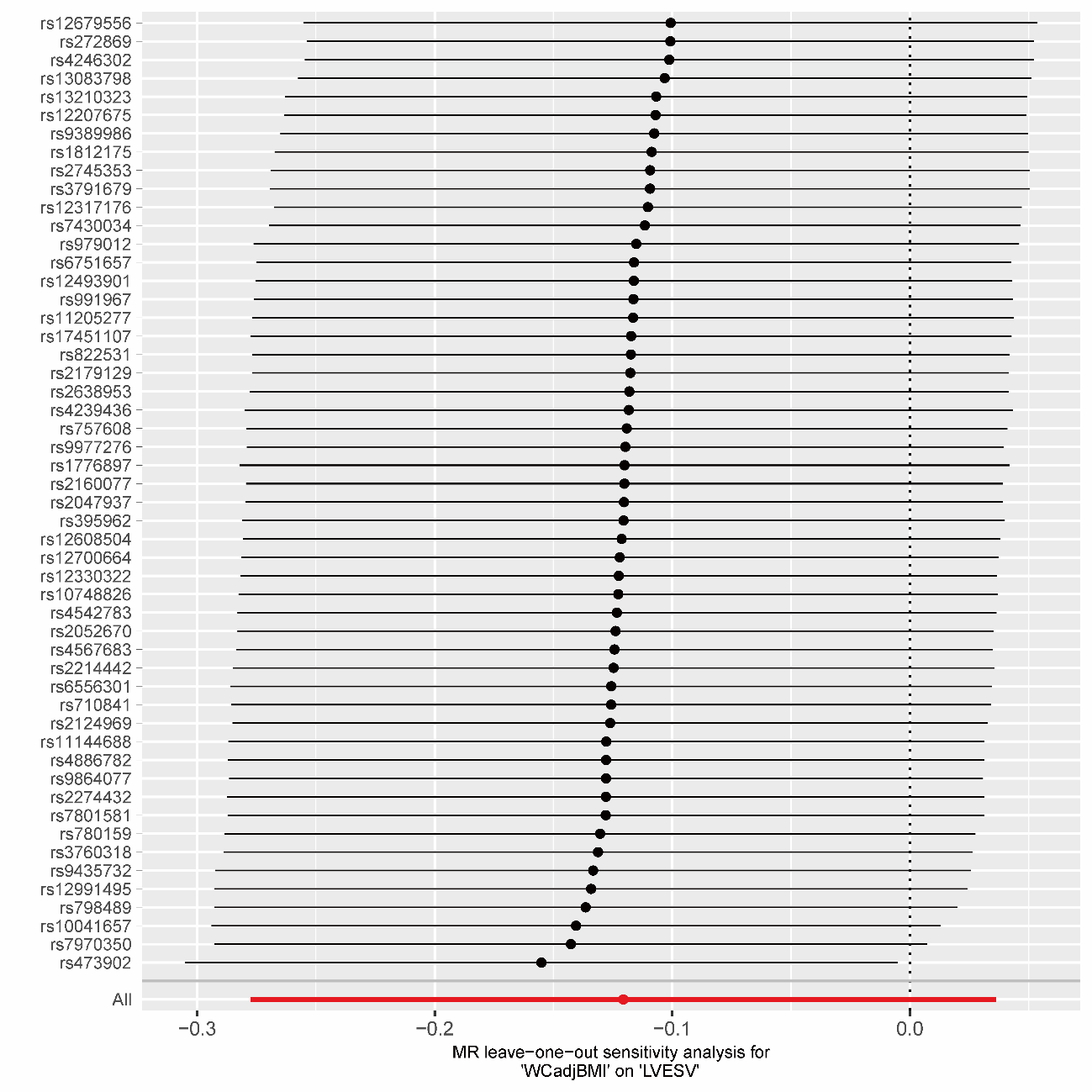


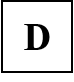


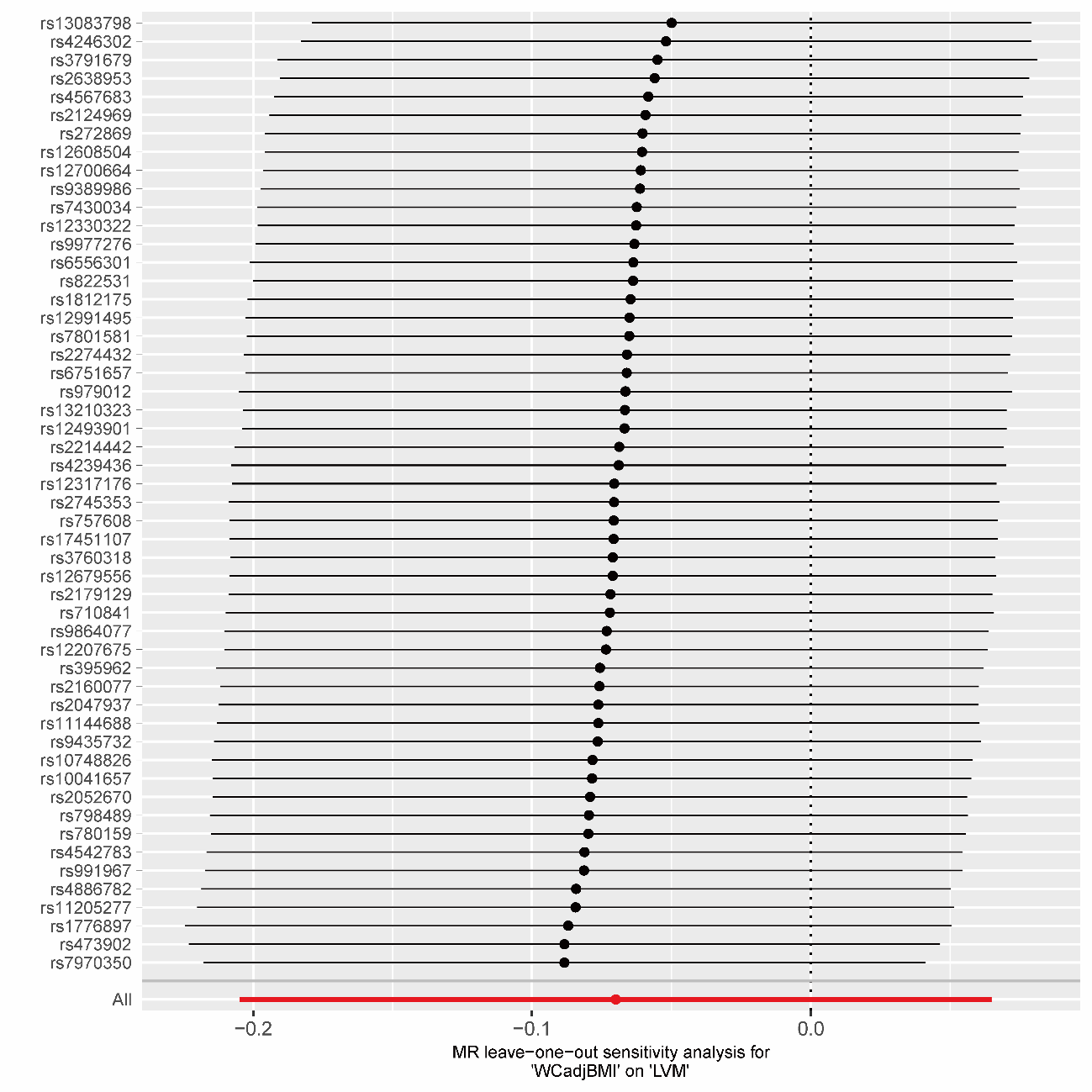


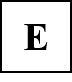


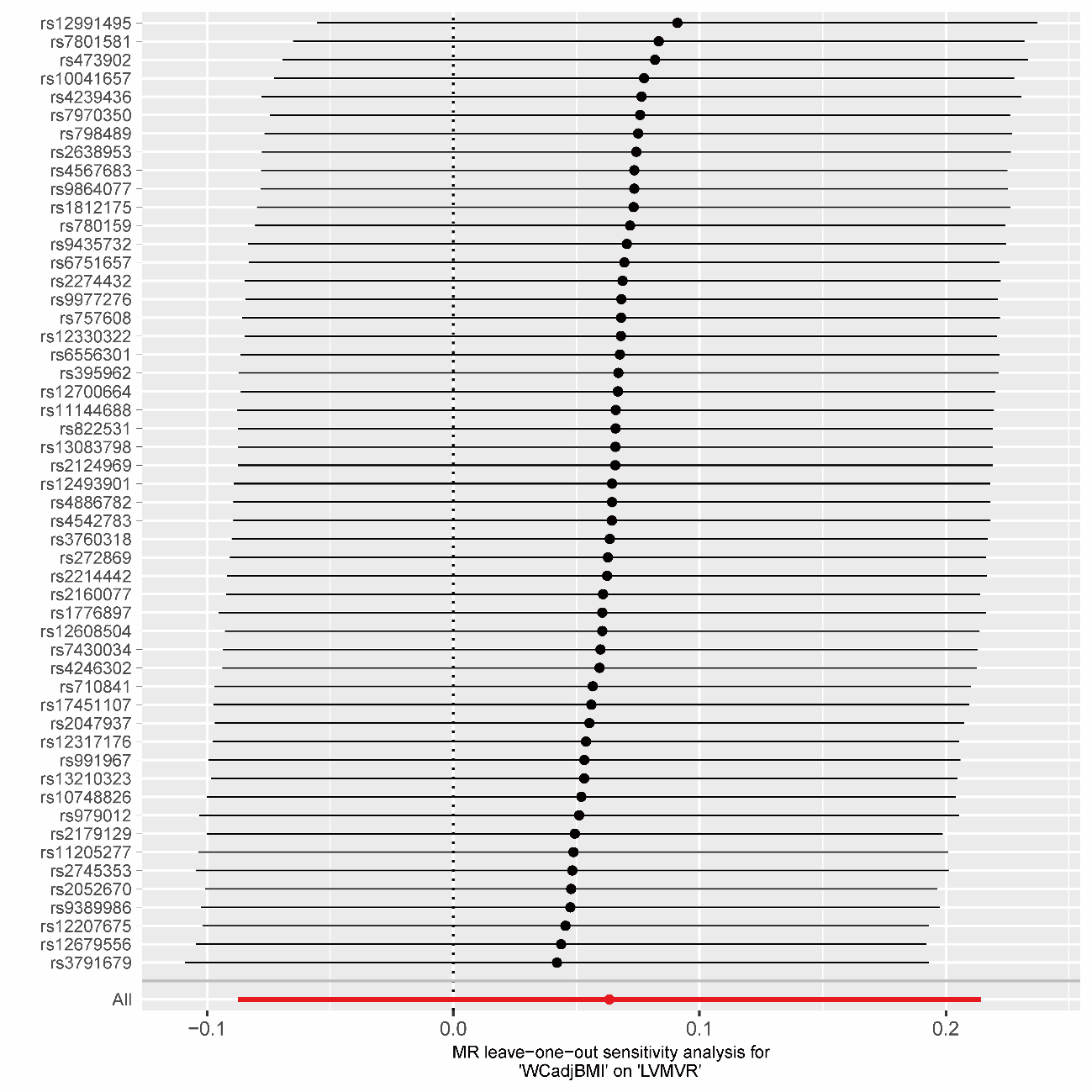


**Figure S5.** Leave-one-out analyses of the association between WCadjBMI and LVEF (A), LVEDV (B), LVESV (C), LVM (D), LVMVR (E). WCadjBMI, waist circumference adjusted for body mass index; LV, left ventricular; LVEF, LV ejection fraction; LVEDV, LV end-diastolic volume; LVESV, LV end-systolic volume; LVM, LV mass; LVMVR, LV mass-to-end-diastolic volume ratio.

**Figure S6.** Leave-one-out analyses of the association between WHRadjBMI and LV parameters.


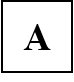

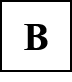


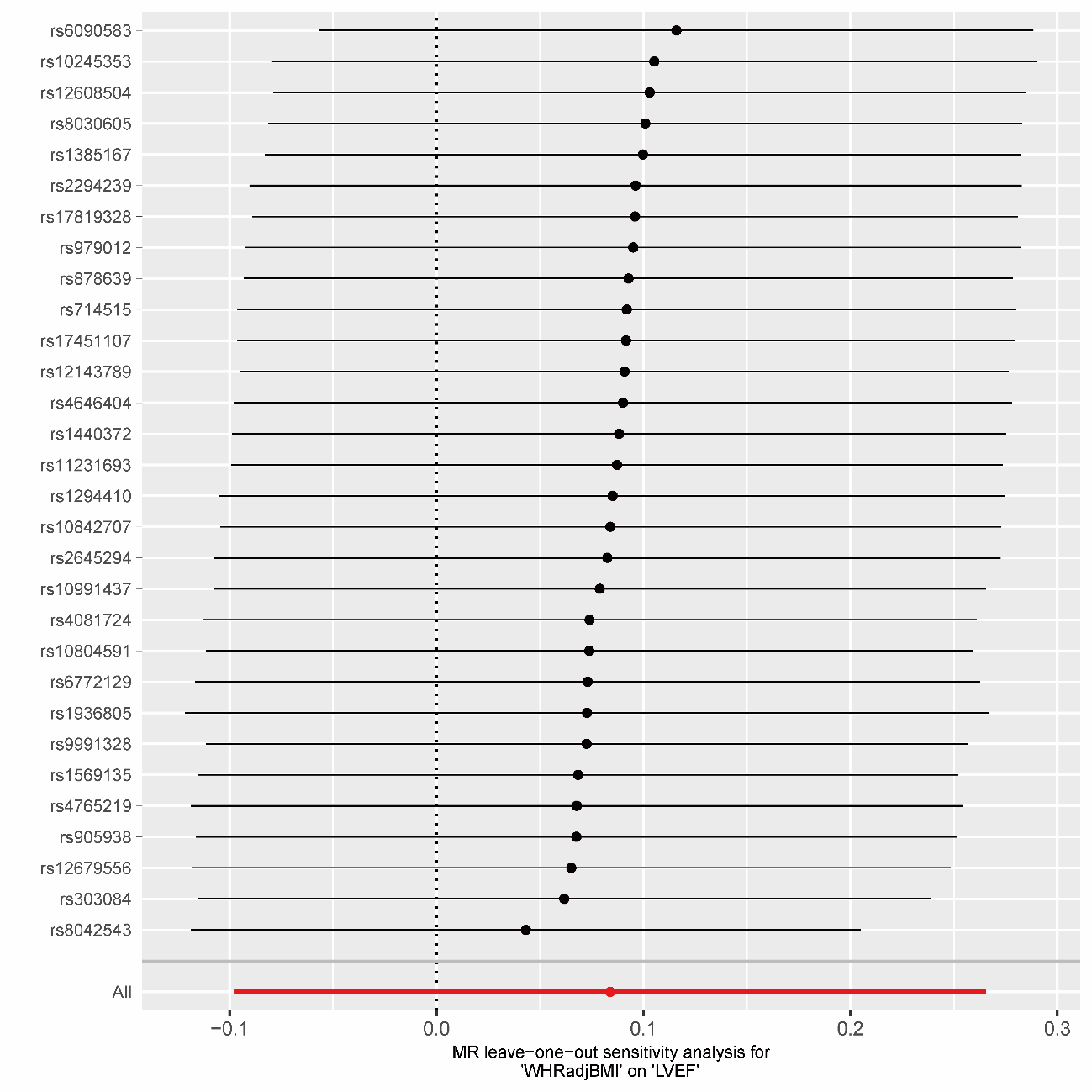


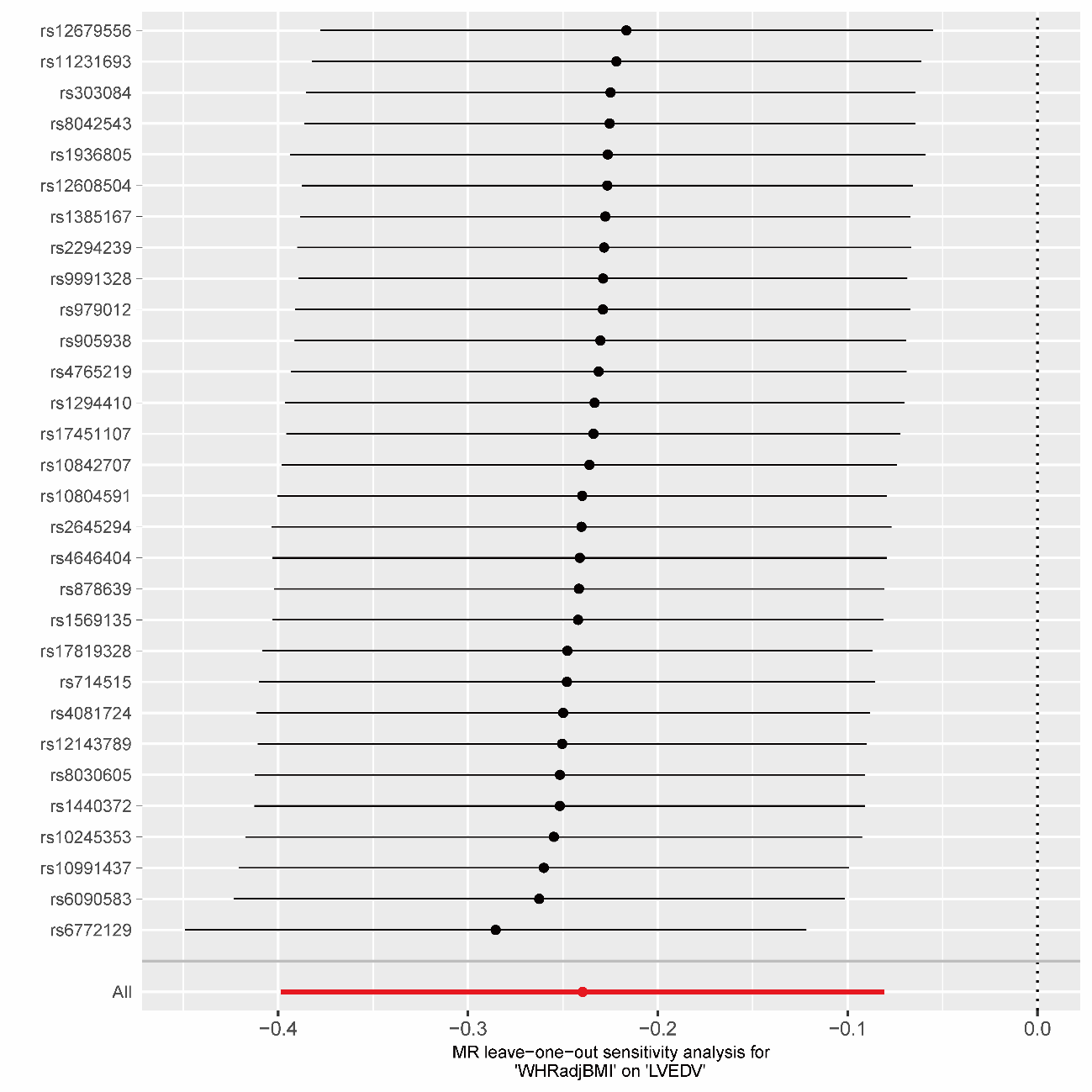


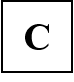

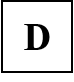


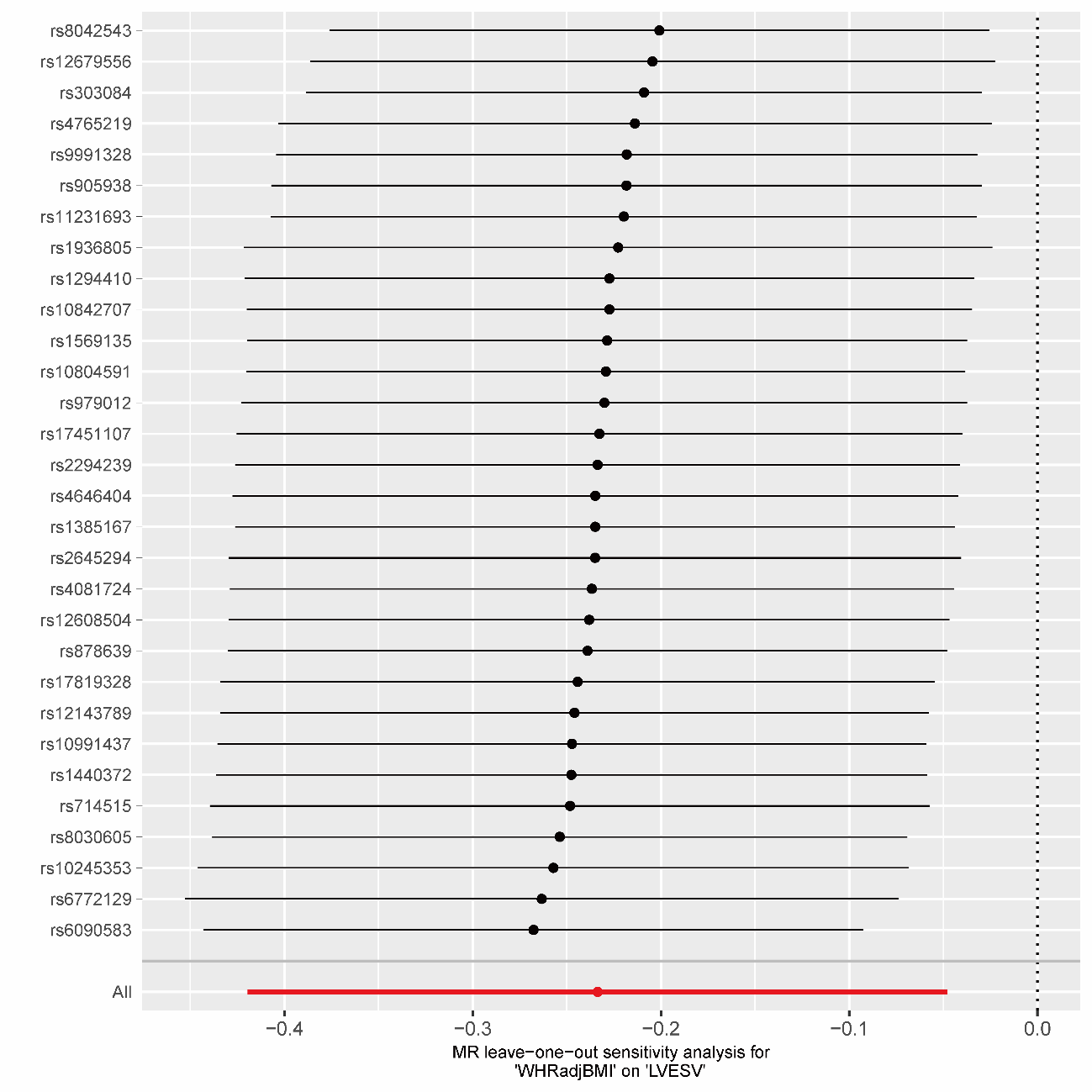


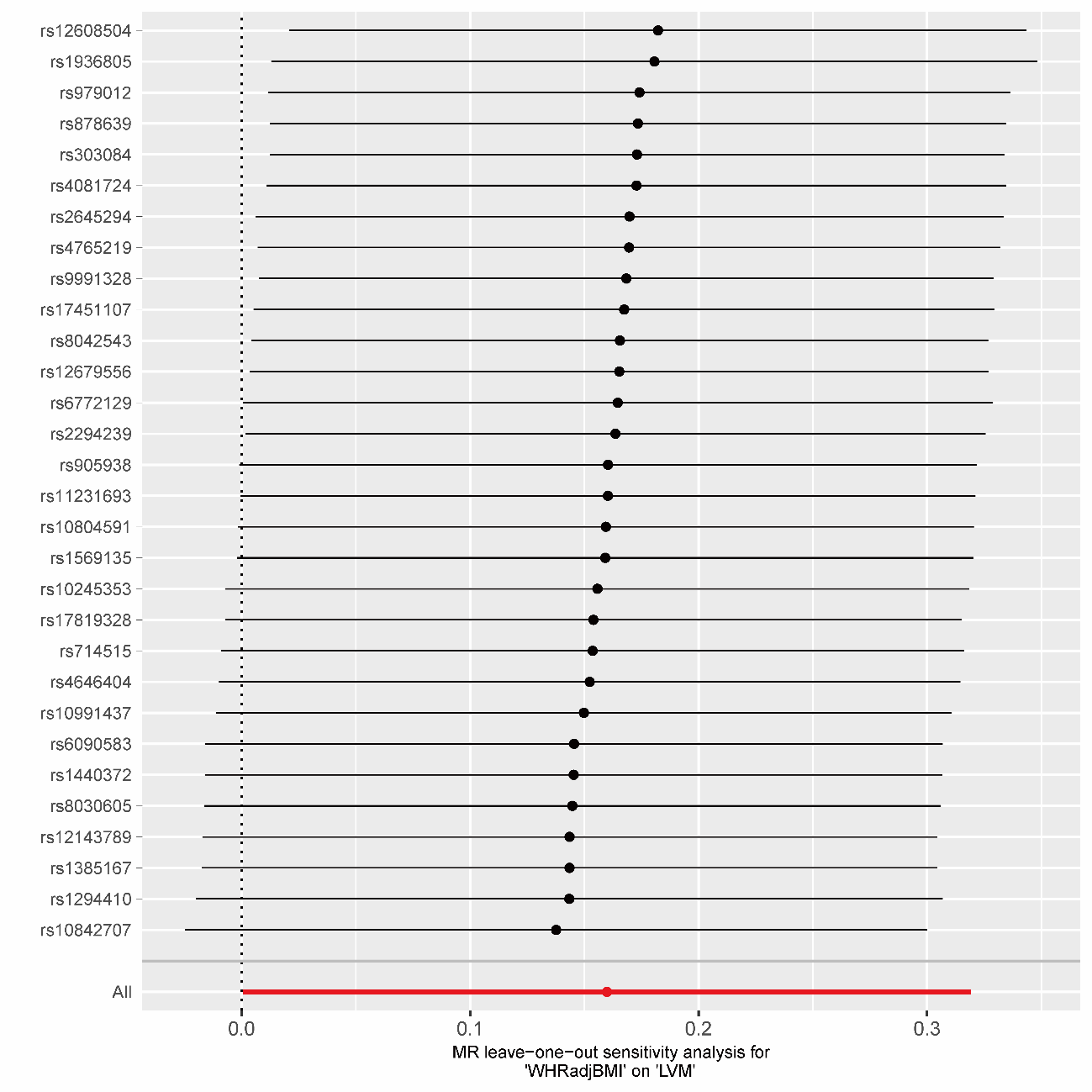


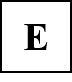


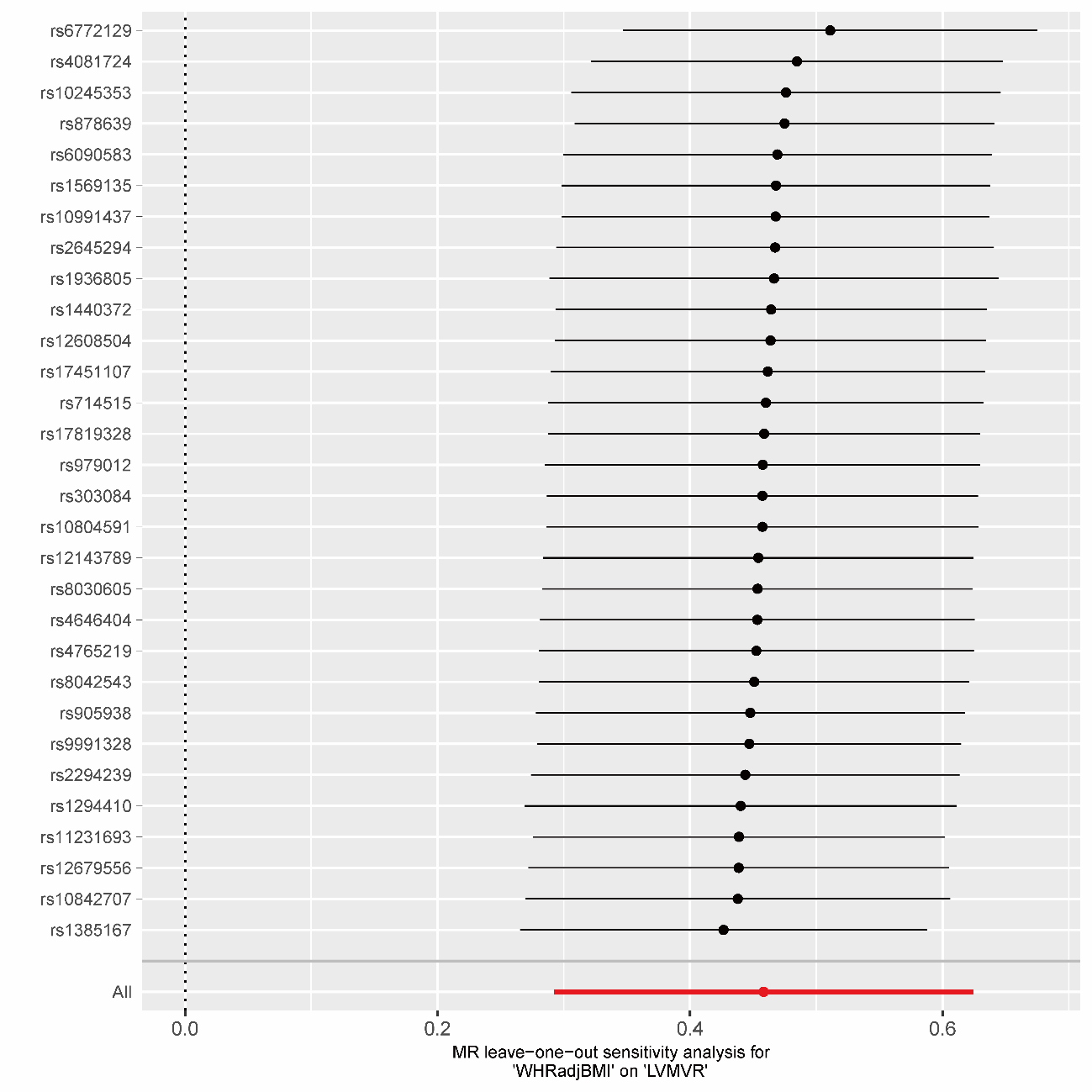


**Figure S6.** Leave-one-out analyses of the association between WHRadjBMI and LVEF (A), LVEDV (B), LVESV (C), LVM (D), LVMVR (E). WHRadjBMI, waist-to-hip ratio adjusted for body mass index; LV, left ventricular; LVEF, LV ejection fraction; LVEDV, LV end-diastolic volume; LVESV, LV end-systolic volume; LVM, LV mass; LVMVR, LV mass-to-end-diastolic volume ratio.
